# Reproductive factors and their association with physical and comprehensive frailty in middle-aged and older women: a large-scale population-based study

**DOI:** 10.1093/hropen/hoae038

**Published:** 2024-06-14

**Authors:** Wenting Hao, Qi Wang, Ruihong Yu, Shiva Raj Mishra, Salim S Virani, Nipun Shrestha, Chunying Fu, Dongshan Zhu

**Affiliations:** Centre for Health Management and Policy Research, School of Public Health, Cheeloo College of Medicine, Shandong University, Jinan, China; NHC Key Lab of Health Economics and Policy Research, Shandong University, Jinan, China; Department of Epidemiology, School of Public Health, Cheeloo College of Medicine, Shandong University, Jinan, China; Pingyin Center for Disease Control and Prevention, Pingyin, Jinan, China; NHMRC Clinical Trials Center, University of Sydney, Sydney, NSW, Australia; Faculty of Medicine and Health, Westmead Applied Research Centre (WARC), University of Sydney, Sydney, NSW, Australia; Michael E DeBakey VA Medical Center, Baylor College of Medicine, Houston, TX, USA; The Aga Khan University, Karachi, Pakistan; Evidence Integration, University of Sydney, Sydney, NSW, Australia; Department of Epidemiology, School of Public Health, Cheeloo College of Medicine, Shandong University, Jinan, China; Department of Epidemiology, School of Public Health, Cheeloo College of Medicine, Shandong University, Jinan, China; Center for Clinical Epidemiology and Evidence-Based Medicine, Shandong University, Jinan, China

**Keywords:** physical frailty, comprehensive frailty, reproductive factors, menopause hormone therapy, age at menopause, parity

## Abstract

**STUDY QUESTION:**

Are women’s reproductive factors associated with physical frailty and comprehensive frailty in middle-age and later life?

**SUMMARY ANSWER:**

Early menarche at <13 years, age at menopause <45 years, surgical menopause, experiencing miscarriage and a shorter reproductive period of <35 years were associated with increased odds of frailty, while having two or three children was related to decreased likelihood of frailty.

**WHAT IS KNOWN ALREADY:**

Evidence has shown that women are frailer than men in all age groups and across different populations, although women have longer lifespans. Female-specific reproductive factors may be related to risk of frailty in women.

**STUDY DESIGN, SIZE, DURATION:**

A population-based cross-sectional study involved 189 898 women from the UK Biobank.

**PARTICIPANTS/MATERIALS, SETTING, METHODS:**

Frailty phenotype and frailty index were used to assess physical frailty and comprehensive frailty (assessed using 38 health indicators for physical and mental wellbeing), respectively. Multivariable logistic regression models were used to estimate odds ratios (ORs) and 95% CI between reproductive factors and likelihood of physical frailty and comprehensive frailty. Restricted cubic spline models were used to test the non-linear associations between them. In addition, we examined the combined effect of categorized age at menopause and menopause hormone therapy (MHT) on frailty.

**MAIN RESULTS AND THE ROLE OF CHANCE:**

There was a J-shape relationship between age at menarche, reproductive period, and frailty; age at menarche <13 years and >16 years, and reproductive period <35 years or >40 years were all associated with increased odds of frailty. There was a negative linear relationship between menopausal age (either natural or surgical) and odds of frailty. Surgical menopause was associated with 30% higher odds of physical frailty (1.34, 1.27–1.43) and 30% higher odds of comprehensive frailty (1.30, 1.25–1.35). Having two or three children was linked to the lowest likelihood of physical frailty (0.48, 0.38–0.59) and comprehensive frailty (0.72, 0.64–0.81). Experiencing a miscarriage increased the odds of frailty. MHT use was linked to increased odds of physical frailty in women with normal age at natural menopause (after 45 years), while no elevated likelihood was observed in women with early natural menopause taking MHT.

**LIMITATIONS, REASONS FOR CAUTION:**

The reproductive factors were self-reported and the data might be subject to recall bias. We lacked information on the types and initiation time of MHT, could not identify infertile women who later became pregnant, and the number of infertile women may be underestimated. Individuals participating in the UK Biobank are not representative of the general UK population, limiting the generalization of our findings.

**WIDER IMPLICATION OF THE FINDINGS:**

The reproductive factors experienced by women throughout their life course can potentially predict frailty in middle and old age. Identifying these reproductive factors as potential predictors of frailty can inform healthcare providers and policymakers about the importance of considering a woman’s reproductive history when assessing their risk for frailty.

**STUDY FUNDING/COMPETING INTEREST(S):**

This work was supported by the National Key Research and Development Program of China (2022YFC2703800), National Natural Science Foundation of China (82273702), Science Fund Program for Excellent Young Scholars of Shandong Province (Overseas) (2022HWYQ-030), Taishan Scholars Project Special Fund (No. tsqnz20221103), and the Qilu Young Scholar (Tier-1) Program (202099000066). All authors have no conflicts of interest to declare.

**TRIAL REGISTRATION NUMBER:**

N/A.

WHAT DOES THIS MEAN FOR PATIENTS? Frailty usually affects people aged 50 years and older. It weakens the body, making it more vulnerable to stress and increasing the risk of falls, disability, hospitalization, and death. Studies show that women are frailer than men, even though they live longer. Female reproductive factors might influence the risk of frailty. This study included 189 898 women from the UK Biobank (a large long-term biobank study in the UK, which is investigating the contributions of genetics and the environmental to the development of disease) to explore how factors, such as age at first period, age at menopause, use of hormone therapy, number of children, infertility, miscarriage, and ages at first and last childbirth, relate to frailty in middle and later life. The findings showed that having a first period before age 13 years, menopause before age 45 years, surgical menopause, miscarriages, and a shorter reproductive period (less than 35 years) increased the risk of frailty, while having two or three children reduced this risk. Using hormone therapy at menopause was linked to higher frailty in women with normal timing of menopause but not in those with early menopause. Our research highlights the importance of considering a woman’s reproductive history when assessing her risk for frailty. This can help healthcare providers and policymakers better understand and manage women’s health as they age.

## Introduction

Frailty, typically occurring in individuals aged 50 years and older (Chen *et al.*, 2014), is a complex ageing-related clinical syndrome characterized by decreased reserves to stressors, affecting approximately 10% of community-dwelling older adults worldwide (Wu, 2023). It carries an increased risk for multiple adverse health-related events including falls, disability, hospitalization, and mortality (Zhang *et al.*, 2020). Previous studies have shown that women are frailer than men in all age groups and across different populations although women have longer lifespans (Kane and Howlett, 2021). Common environmental, social, lifestyle, psychological, or evolutionary factors cannot fully explain the sex difference (Gordon *et al.*, 2017; Gordon and Hubbard, 2020; Park and Ko, 2021). The generally smaller stature of women than men may correlate with lower muscle mass, a key factor in frailty (Nguyen *et al.*, 2022). In addition, female-specific reproductive factors may be related to risk of frailty in women.

Menarche and menopause mark the onset and cessation of ovarian activity, respectively, and are accompanied by changes in endogenous estrogen level (Collaborative Group on Hormonal Factors in Breast Cancer, 2012). Age of menarche or menopause has been related to multiple health outcomes (Shen *et al.*, 2019; Hu *et al.*, 2021; Li *et al.*, 2021). The associations between age of menarche, age at menopause, reproductive duration (from menarche to menopause), and risk of frailty remain unclear. Only one study examined the association between age at menarche and frailty, but no relationship between them was observed (Mamalaki *et al.*, 2019). Several studies have shown that later menopause and longer reproductive span were associated with a lower risk of frailty (Lee *et al.*, 2020; Kojima *et al.*, 2022b). A recent prospective study involving women in the UK found that earlier menopause was associated with higher risk of incident frailty, while no association was found between later menopause and frailty (Kojima *et al.*, 2022a). Also, different types of menopause (natural menopause or surgical menopause) might be linked to different frailty risks. The association between surgical menopause and frailty risk has not been well examined (Gomes *et al.*, 2018; Kojima *et al.*, 2022b). In addition, evidence has shown that age at first birth and number of pregnancies were related to risk of frailty in later life (Gomes *et al.*, 2018; Kojima *et al.*, 2020; Guo *et al.*, 2023). Previous studies focused on the association of reproductive factors with risk of frailty in older women, aged over 60 or 65 years (Gomes *et al.*, 2018; Velez *et al.*, 2019; Lee *et al.*, 2020; Kojima *et al.*, 2022a). Further, no study has examined the relationship between age at last birth and risk of frailty.

We aimed to examine the relationships between a series of female-specific reproductive factors (age at menarche, age at natural menopause, age at surgical menopause, menopause hormone therapy (MHT), reproductive period, number of children, age at the first child’s birth, age at the last child’s birth) and frailty in middle age and later life.

## Materials and methods

### Study and population

**Figure 1.**
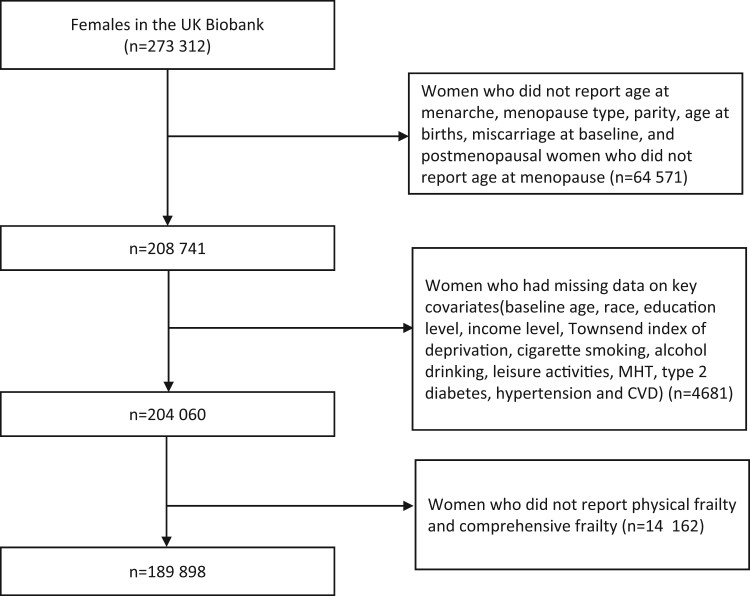
**Flow diagram of participant selection from the UK Biobank for a study of reproductive factors and frailty in middle-aged and older women**. MHT, menopause hormone therapy; CVD, cardiovascular disease.

The UK Biobank is a large population-based prospective cohort study to investigate the role of comprehensive exposures in health and diseases. From April 2006 to December 2010, the UK Biobank recruited over 500 000 participants (40–69 years old) from 22 assessment centers across England, Wales, and Scotland (Sudlow *et al.*, 2015; Gong *et al.*, 2021). At enrolment, UK Biobank collected participants’ data on sociodemographic, lifestyle, and health-related factors through touch-screen tests, questionnaires, physical measurements, and biological samples. Participants gave informed consent for data linkage to national hospital inpatient admissions, cancer registrations, and death registrations. UK Biobank has approval from the North West Multicenter Research Ethics Committee (https://www.ukbiobank.ac.uk/learn-more-about-uk-biobank/about-us/ethics). This research was conducted under UK Biobank application number 68369. A cross-sectional analysis was adopted based on women who had information on reproductive factors and frailty at baseline and had no missing key covariates. A total of 189 898 women were included (Fig. 1).

### Exposure variables

In the present study, age at menarche was categorized as <11, 12, 13, 14, 15, and ≥16 years (late menarche). Parity is the number of live births and was classified into six categories: 0, 1, 2, 3, 4, and ≥5. Timing of birth included age at the first child’s birth (categorized as 13–21, 22–25, 26–29, and ≥30 years) and age at the last child’s birth (categorized as 13–25, 26–29, 30–33, and ≥34 years). Infertility and miscarriage experiences were classified into yes or no. Information on miscarriage was collected via a self-reported question ‘Have you ever had any stillbirths, spontaneous miscarriages, or terminations?’ with optional responses of ‘yes’ or ‘no’. Infertility was defined by the International Classification of Diseases-10 code N97 (Female infertility). Natural menopause was defined as the cessation of menstruation for 12 consecutive months without prior hysterectomy and/or oophorectomy. Surgical menopause was defined as the removal of both ovaries (bilateral oophorectomy) before natural menopause. Hysterectomy was defined as removal of the uterus before natural menopause without bilateral oophorectomy, and age of hysterectomy was categorized as ≤40, 41–45, 46–50, 51–55, and >55 years. Age at natural menopause was categorized as pre-menopause, perimenopause, ≤40 (premature), 41–45 (early), 46–50, 51–55, and >55 years (late menopause) (Hao *et al.*, 2023). Perimenopausal women were defined as those who were experiencing irregular menstrual cycles (<14 days or >90 days), or those who had not menstruated in the past 3 months but had menstruated within the last 12 months. The reproductive period was defined as the interval between the age at menarche and the age at menopause and was classified as <30, 30–34, 35–39, and ≥40 years.

### Outcomes

Physical frailty and comprehensive frailty were assessed at baseline (2006–2010). We used the two most common frailty measures, frailty phenotype (FP), and frailty index (FI), to assess physical frailty and comprehensive frailty, respectively. FP criteria were derived by Fried *et al.* (2001) in the Cardiovascular Health Study and were later validated in the UK Biobank data (Hanlon *et al.*, 2018). FP was characterized by five clinical indicators: unintentional weight loss, exhaustion, low physical activity, slow walking speed, and low grip strength (weakness). Participants were classified as frail if they exhibited three or more indicators, pre-frail if they showed one or two, and robust if they had none. Detailed definitions of each clinical indicator of FP are provided in Supplementary Table S1. The FI metrics represent a non-weighted sum of accumulated health-related deficits, based on 38 deficits (Rockwood and Mitnitski, 2007) that had previously been validated using baseline data from the UK Biobank (Williams *et al.*, 2019). FI was derived by dividing the number of deficits presented by each individual by the total number of possible deficits. It was a value between 0 and 1, and a higher value indicated a greater degree of frailty. Participants were classified as being robust (<0.08), pre-frail (0.08–0.25), or frail (≥0.25) (Mutz *et al.*, 2022). The detailed items and scoring of FI are described in Supplementary Table S2. Frailty was divided into non-frailty (robust and pre-frail) and frailty, and non-frailty was taken as the reference.

### Covariates

We included the following factors in the analyses as covariates according to evidence from previous studies (Gomes *et al.*, 2018; Velez *et al.*, 2019; Kojima *et al.*, 2020): age at baseline, race, education level, income level, Townsend deprivation index, BMI, cigarette smoking, alcohol drinking, leisure activities, MHT status, cardiovascular disease (CVD), diabetes, and hypertension status. Race was categorized as White and non-White. Years of education were categorized as ≤10, 11–12, and >12 years. Income level was divided into four categories of level 1 (<£18 000), level 2 (£18 000 to £30 999), level 3 (£31 000 to £51 999), and level 4 (>£52 000). In addition, we also included the Townsend deprivation index (an area-level socioeconomic status), which represents a comprehensive score of four key variables: unemployment, household overcrowding, non-car ownership, and non-home ownership. The Townsend deprivation index was divided into quartiles: Q1 (least deprivation) to Q4 (most deprivation), with a higher score representing higher levels of deprivation (Blane *et al.*, 1987; Ye *et al.*, 2021). BMI was classified as <18.5 kg/m^2^, 18.5–24.9 kg/m^2^, 25–29.9 kg/m^2^, and ≥30 kg/m^2^. Smoking was classified as current smoking, former smoking, and never smoking. Alcohol status was categorized as current drinking, former drinking, and never drinking. Leisure activities were assessed through participants’ choices of six activities and were categorized as none, one, and two or more. MHT status was collected at baseline through a question, ‘Have you ever used HRT?’ and was classified as user or non-user. CVD, hypertension, or diabetes status was dichotomized as present or absent based on self-report at baseline. Detailed information on covariates collection and definitions is listed in Supplementary Table S3.

### Statistical analysis

Continuous and categorical variables were presented as mean±SD and number (percentage), respectively. Multivariable logistic regression models were used to estimate odds ratios (ORs) and 95% CI between reproductive factors and frailty. Covariates were adjusted sequentially. In Model 1, age at baseline was adjusted. In Model 2, potential confounders were further adjusted, including race, education levels, income levels, Townsend index of deprivation, cigarette smoking, alcohol drinking, leisure activities, and MHT. In model 3, CVD, hypertension, and diabetes were further adjusted for the association with physical frailty. In addition, in assessing the link between individual reproductive factors and frailty, reproductive factors were adjusted for each other, as follows in detail: for the relationship between age at menopause and frailty, age at menarche and number of live births were further adjusted in model 3; for the relationship between number of children and frailty, age at menarche and age at menopause were further adjusted; for the relationship between age at first or last birth and frailty, age at menarche and number of children were further adjusted; and for the relationship between miscarriage, infertility and frailty, age of menarche, and age at menopause were further adjusted. Multiple imputation techniques were used to impute missing covariates as a sensitivity analysis. We also stratified the analyses by age (<60 years and ≥60 years).

To quantify the dose–response relationships, we used restricted cubic spline models to test the non-linear associations between reproductive factors and frailty. We also stratified the dose–response relationships by race (White, Asian, and Black). In addition, we examined the combined effect of categorized age at menopause and MHT on frailty. All analyses were performed in SAS 9.4 (SAS Institute, Cary, NC, USA) and R v4.2.1 (R Core Team, Vienna, Austria).

## Results

### Participant characteristics

The mean age at baseline was 56.2 (SD 7.9) years, with a range from 40 to 71 years old. There were 10 810 (5.7%) women with physical frailty and 38 444 (20.2%) with comprehensive frailty. Compared to women without frailty, women with physical frailty or comprehensive frailty were more likely to have less income, be current smokers, have no leisure activities, and have diabetes, CVD, and hypertension (Table 1). Also, women with frailty had a higher percentage of having menarche before age 11 years, of having menopause before age 45 years, and of having shorter reproductive duration (<30 years) (Table 2). Compared to women who were included, those who were excluded because of missing data had a similar age at baseline to those who were included (mean age 56.7 vs 56.2 years, respectively) (Supplementary Table S4).

**Table 1.** Baseline characteristics of 189 898 women from the UK Biobank for assessment of physical frailty and comprehensive frailty.

Characteristics	Physical frailty		Comprehensive frailty	
	No-frailty	Frailty	No-frailty	Frailty
**N (%)**	179 088 (94.3)	10 810 (5.7)	151 454 (79.8)	38 444 (20.2)
**Age (years, mean±SD)**	56.1 ± 8.0	57.3 ± 7.7	55.8 ± 8.0	57.6 ± 7.6
**Age (years, categories)**				
<60	106 268 (59.3)	5968 (55.2)	92 185 (60.9)	20 051 (52.2)
≥60	72 820 (40.7)	4842 (44.8)	59 269 (39.1)	18 393 (47.8)
**Race**				
White	170 969 (95.5)	9820 (90.8)	143 995 (95.1)	36 794 (95.7)
Asian	2880 (1.6)	409 (3.8)	2785 (1.8)	504 (1.3)
Black	2318 (1.3)	277 (2.6)	2102 (1.4)	493 (1.3)
Others	2921 (1.6)	304 (2.8)	2572 (1.7)	653 (1.7)
**Education level (years)**				
≤10	84 564 (47.2)	6588 (61.0)	72 499 (47.9)	18 653 (48.5)
11–12	22 926 (12.8)	1224 (11.3)	19 309 (12.8)	4841 (12.6)
>12	71 598 (40.0)	2998 (27.7)	59 646 (39.3)	14 950 (38.9)
**Income level (£)**				
<18000	41 237 (23.0)	4096 (37.9)	33 764 (22.3)	11 569 (30.1)
18000–30999	44 943 (25.1)	2633 (24.4)	37 797 (25.0)	9779 (25.4)
31 000–51 999	46 373 (25.9)	2156 (19.9)	39 511 (26.0)	9018 (23.5)
≥52 000	46 535 (26.0)	1925 (17.8)	40 382 (26.7)	8078 (21.0)
**Townsend deprivation index**				
Ql: least deprived	44 930 (25.1)	1755 (16.2)	38 141 (25.2)	8544 (22.2)
Q2	44 587 (24.9)	2042 (18.9)	37 867 (25.0)	8762 (22.8)
Q3	45 262 (25.3)	2607 (24.1)	38 266 (25.3)	9603 (25.0)
Q4: most deprived	44 309 (24.7)	4406 (40.8)	37 180 (24.5)	11 535 (30.0)
**BMI**				
<18.5 kg/m^2^	1387 (0.8)	92 (0.9)	1176 (0.8)	303 (0.8)
18.5–24.9 kg/m^2^	74 341 (41.5)	1999 (18.5)	63 578 (42.0)	12 762 (33.2)
25.0–29.9 kg/m^2^	65 636 (36.7)	3194 (29.6)	55 227 (36.4)	13 603 (35.4)
≥30 kg/m^2^	37 724 (21.0)	5525 (51.0)	31 473 (20.8)	11 776 (30.6)
**Cigarette smoking**				
Never smoked	107 225 (59.9)	5847 (54.1)	91 674 (60.5)	21 398 (55.7)
Former smoker	57 007 (31.8)	3409 (31.5)	47 199 (31.2)	13 217 (34.3)
Current smoker	14 856 (8.3)	1554 (14.4)	12 581 (8.3)	3829 (10.0)
**Alcohol drinking**				
Never a drinker	8762 (4.9)	1218 (11.3)	7522 (5.0)	2458 (6.4)
Former drinker	5584 (3.1)	883 (8.2)	4083 (2.7)	2384 (6.2)
Current drinker	164 742 (92.0)	8709 (80.5)	139 849 (92.3)	33 602 (87.4)
**No. of leisure activities**				
0	47 909 (26.8)	4581 (42.4)	40 790 (26.9)	11 700 (30.4)
1	75 971 (42.4)	4220 (39.0)	64 508 (42.6)	15 683 (40.8)
≥2	55 208 (30.8)	2009 (18.6)	46 156 (30.5)	11 061 (28.8)
**MHT**				
No	113 906 (63.6)	5937 (54.9)	98 972 (65.4)	20 871 (54.3)
Yes	65 182 (36.4)	4873 (45.1)	52 482 (34.6)	17 573 (45.7)
**Diabetes** *				
No	173 770 (97.0)	9510 (88.3)	147 409 (97.3)	35 871 (93.5)
Yes	5318 (3.0)	1300 (11.7)	4045 (2.7)	2573 (6.5)
**CVD** *				
No	171 786 (95.8)	9218 (85.9)	146 641 (96.7)	34 363 (90.0)
Yes	7302 (4.2)	1592 (14.1)	4813 (3.3)	4081 (10.0)
**Hypertension** *				
No	137 516 (76.5)	6197 (58.5)	118 648 (78.7)	25 065 (66.6)
Yes	41 572 (23.5)	4613 (41.5)	32 806 (22.3)	13 379 (33.4)

Data are n (%).

* Prevalence of diabetes, CVD, and hypertension was adjusted using the age distribution of all women in the UK Biobank as the standard age distribution.

Q1–Q4, Quartile 1 ∼ Quartile 4; MHT, menopausal hormone therapy; CVD, cardiovascular disease.

**Table 2.** Reproductive characteristics of 189 898 women from the UK Biobank for assessment of physical frailty and comprehensive frailty.

Reproductive characteristics	Physical frailty		Comprehensive frailty	
	No-frailty	Frailty	No-frailty	Frailty
**Age at menarche (years)**				
<11	34 530 (19.3)	2794 (25.8)	28 842 (19.0)	8482 (22.1)
12	34 295 (19.2)	1977 (18.3)	29 019 (19.2)	7253 (18.9)
13	44 673 (24.9)	2310 (21.4)	37 886 (25.0)	9097 (23.7)
14	35 984 (20.1)	1882 (17.4)	30 619 (20.2)	7247 (18.8)
15	19 540 (10.9)	1152 (10.7)	16 603 (11.0)	4089 (10.6)
≥16	10 066 (5.6)	695 (6.4)	8485 (5.6)	2276 (5.9)
**Menopause type**				
Pre-menopause	34 500 (19.0)	387 (3.6)	6198 (4.1)	1297 (3.3)
Perimenopause	7108 (4.0)	1530 (14.2)	31 010 (20.5)	5020 (13.1)
Natural menopause	120027 (67.2)	7112 (65.8)	100 550 (66.4)	26 589 (69.2)
Surgical menopause	17 453 (9.8)	1781 (16.4)	13 696 (9.0)	5538 (14.4)
**Age at natural menopause (years)**				
≤40	3799 (2.4)	401 (4.5)	3012 (2.2)	1188 (3.6)
41–45	11 927 (7.4)	907 (10.0)	9806 (7.1)	3028 (9.2)
46–50	40 554 (25.1)	2478 (27.4)	34 061 (24.7)	8971 (27.2)
51–55	51 815 (32.0)	2617 (29.0)	43 696 (31.7)	10 736 (32.6)
>55	11 932 (7.4)	709 (7.8)	9975 (7.2)	2666 (8.1)
Perimenopause	7108 (4.4)	1530 (17.0)	31 010 (22.6)	5020 (15.4)
Pre-menopause	34 500 (21.3)	387 (4.3)	6198 (4.5)	1297 (3.9)
**Age at surgical menopause (years)** ^a^				
≤40	2918 (16.7)	433 (24.3)	2185 (16.0)	1166 (21.1)
41–45	3395 (19.5)	352 (19.8)	2680 (19.6)	1067 (19.3)
46–50	5249 (30.1)	453 (25.4)	4191 (30.6)	1511 (27.3)
51–55	3128 (17.9)	268 (15.1)	2464 (18.0)	932 (16.8)
>55	2763 (15.8)	275 (15.4)	2176 (15.8)	862 (15.5)
**Reproductive period (years)**				
<30	10 961 (8.0)	1157 (13.0)	8629 (7.6)	3489 (10.9)
30–34	22 488 (16.4)	1674 (18.8)	18 579 (16.3)	5583 (17.4)
35–39	57 344 (41.6)	3250 (36.6)	47 984 (42.0)	12 610 (39.3)
≥40	46 685 (34.0)	2810 (31.6)	39 051 (34.1)	10 444 (32.4)
**Parity**				
0	34 318 (19.2)	2091 (19.3)	28 424 (18.8)	7985 (20.8)
1	24 034 (13.4)	1643 (15.2)	20 183 (13.3)	5494 (14.3)
2	79 257 (44.3)	4135 (38.3)	67 537 (44.6)	15 855 (41.2)
3	31 207 (17.4)	1872 (17.3)	26 514 (17.5)	6565 (17.1)
4	7881 (4.4)	690 (6.4)	6731 (4.4)	1840 (4.8)
≥5	2391 (1.3)	379 (3.5)	2065 (1.4)	705 (1.8)
**Age at first birth (years)**				
No birth	34 318 (19.2)	2091 (19.3)	28 424 (18.8)	7985 (20.7)
13–21	26 451 (14.8)	2739 (25.4)	22 302 (14.7)	6888 (17.9)
22–25	41 033 (22.9)	2631 (24.3)	34 602 (22.9)	9062 (23.6)
26–29	42 545 (23.7)	1858 (17.2)	36 225 (23.9)	8178 (21.3)
≥30	34 741 (19.4)	1491 (13.8)	29 901 (19.7)	6331 (16.5)
**Age at last birth (years)**				
No birth	34 318 (19.2)	2091 (19.3)	28 424 (18.8)	7985 (20.8)
13–25	25 306 (14.1)	2197 (20.4)	21 179 (14.0)	6324 (16.4)
26–29	39 741 (22.2)	2417 (22.4)	33 514 (22.1)	8644 (22.5)
30–33	40 374 (22.5)	2122 (19.6)	34 446 (22.7)	8050 (20.9)
≥34	39 349 (22.0)	1983 (18.3)	33 891 (22.4)	7441 (19.4)
**Miscarriage**				
No	120 354 (67.2)	6904 (63.9)	102 047 (67.4)	25 211 (65.6)
Yes	58 734 (32.8)	3906 (36.1)	49 407 (32.6)	13 233 (34.4)
**Infertility**				
No	178 148 (99.5)	10 774 (99.7)	150 626 (99.5)	38 296 (99.6)
Yes	940 (0.5)	36 (0.3)	828 (0.5)	148 (0.4)
**Age of hysterectomy (years)** ^b^				
≤40	3225 (18.8)	489 (28.0)	2389 (17.7)	1325 (24.4)
41–45	3345 (19.5)	348 (19.9)	2650 (19.7)	1043 (19.2)
46–50	5106 (29.7)	429 (24.6)	4092 (30.3)	1443 (26.6)
51–55	2926 (17.0)	241 (13.8)	2313 (17.1)	854 (15.7)
>55	2572 (15.0)	240 (13.7)	2047 (15.2)	765 (14.1)

a 19 234 women who experienced surgical menopause were involved.

b 18 921 women who experienced hysterectomy were involved.

Data are n (%).

### Age at menarche and frailty

**Figure 2.**
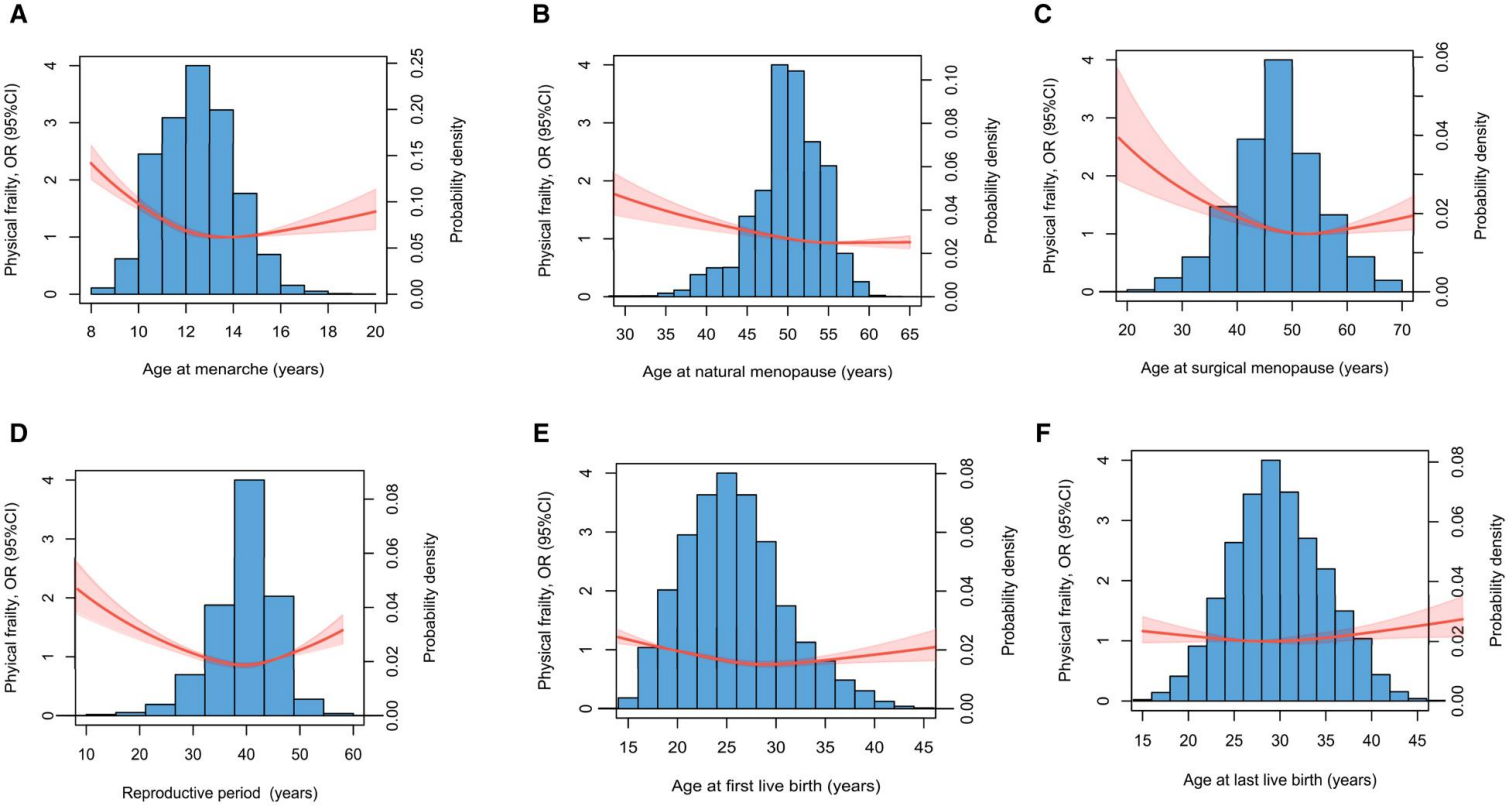
**Dose–response associations between reproductive factors and physical frailty in women in the UK Biobank (N = 189 898)**. OR, odds ratio.

**Figure 3.**
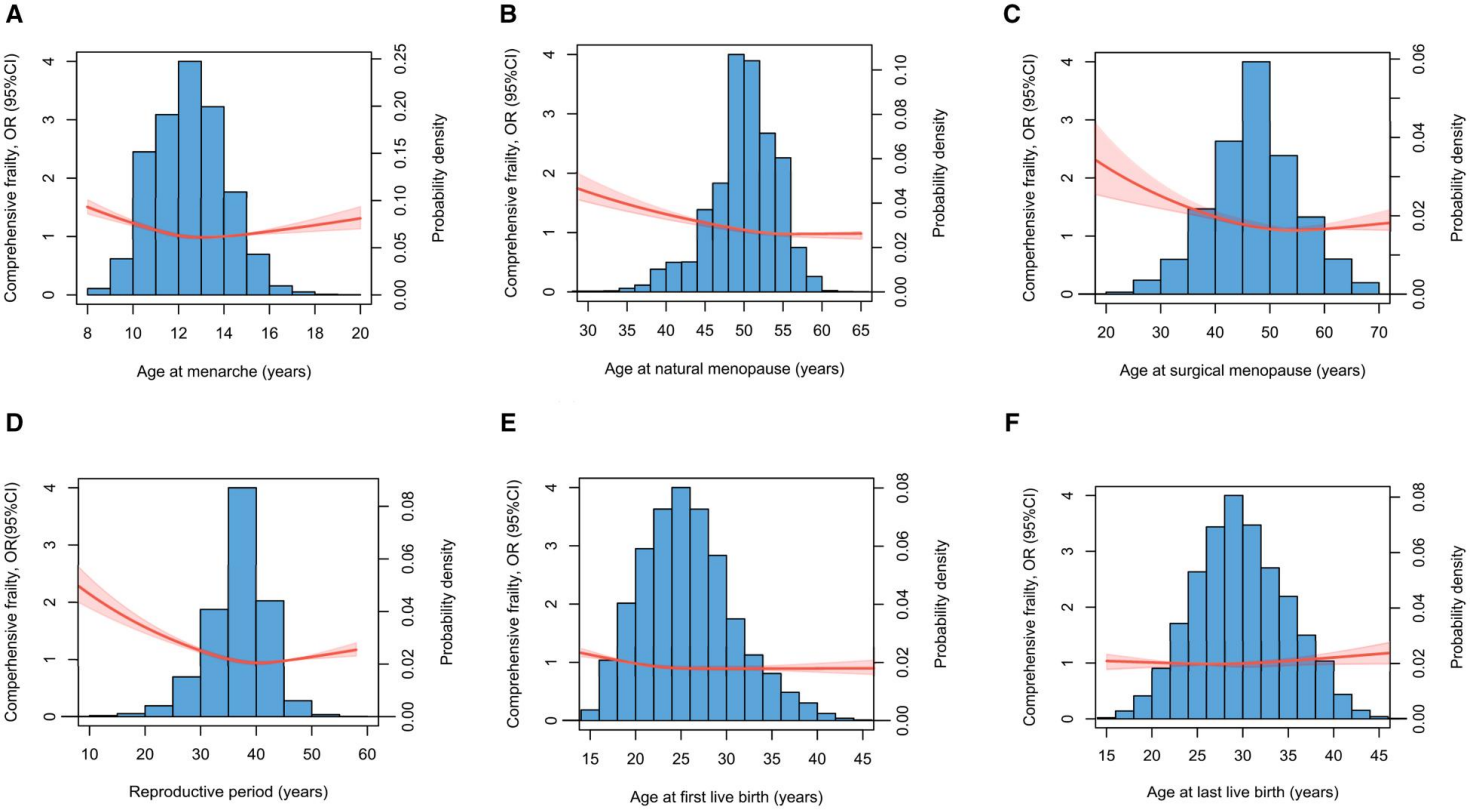
**Dose–response associations between reproductive factors and comprehensive frailty in women in the UK Biobank (N = 189 898)**. OR, odds ratio.

Compared to women who experienced menarche at age 14 years, women who had menarche at age <11 years, 12 years, and >16 years had increased odds of physical frailty, with OR (95% CI) of (1.41, 1.32–1.50), (1.12, 1.04–1.19), and (1.14, 1.04–1.25), respectively, and similar associations were also found with comprehensive frailty, with OR (95% CI) of (1.13, 1.09–1.17), (1.03, 1.00–1.07), and (1.13, 1.07–1.19), respectively (Table 3). Restricted cubic spline models showed that there was an approximately J-shape relationship between continuous age at menarche and physical frailty (Fig. 2A) and comprehensive frailty (Fig. 3A).

**Table 3.** Odd ratios and 95% CI between reproductive characteristics and physical frailty and comprehensive frailty in women from the UK Biobank.

Reproductive characteristics	Physical frailty			Comprehensive frailty	
	Model 1	Model 2	Model 3	Model 1	Model 2
**Age at menarche (years)**					
<11	1.55 (1.46, 1.64)	1.51 (1.42, 1.60)	1.41 (1.32, 1.50)	1.25 (1.20, 1.29)	1.13 (1.09, 1.17)
12	1.11 (1.04, 1.18)	1.15 (1.07, 1.23)	1.12 (1.04, 1.19)	1.06 (1.03, 1.10)	1.03 (1.00, 1.07)
13	0.99 (0.93, 1.06)	1.04 (0.98, 1.11)	1.04 (0.97, 1.11)	1.03 (0.99, 1.06)	1.03 (0.99, 1.06)
14	1	1	1	1	1
15	1.13 (1.05, 1.22)	1.04 (0.96, 1.12)	1.04 (0.97, 1.13)	1.04 (1.00, 1.09)	1.04 (0.99, 1.08)
≥16	1.33 (1.22, 1.45)	1.14 (1.04, 1.25)	1.14 (1.04, 1.25)	1.15 (1.09, 1.21)	1.13 (1.07, 1.19)
**Menopause type**					
Pre-menopause	0.77 (0.73, 0.82)	0.78 (0.73, 0.83)	0.88 (0.83, 0.94)	0.67 (0.65, 0.70)	0.75 (0.72, 0.77)
Perimenopause	0.95 (0.85, 1.06)	0.97 (0.86, 1.08)	1.04 (0.93, 1.16)	0.87 (0.82, 0.93)	0.94 (0.88, 1.00)
Natural menopause	1	1	1	1	1
Surgical menopause	1.72 (1.63, 1.81)	1.45 (1.37, 1.53)	1.34 (1.27, 1.43)	1.52 (1.46, 1.57)	1.30 (1.25, 1.35)
**Age at natural menopause (years)**					
≤40	1.73 (1.55, 1.93)	1.37 (1.22, 1.54)	1.32 (1.17, 1.48)	1.50 (1.39, 1.61)	1.32 (1.22, 1.42)
41–45	1.24 (1.15, 1.34)	1.11 (1.02, 1.20)	1.09 (1.00, 1.18)	1.17 (1.11, 1.22)	1.10 (1.04, 1.15)
46–50	1	1	1	1	1
51–55	0.83 (0.78, 0.87)	0.92 (0.87, 0.98)	0.92 (0.87, 0.98)	0.93 (0.90, 0.96)	0.96 (0.93, 0.99)
>55	0.95 (0.88, 1.04)	1.06 (0.97, 1.15)	1.02 (0.93, 1.11)	0.97 (0.90, 1.02)	0.97 (0.92, 1.02)
Perimenopause	0.93 (0.83, 1.04)	0.96 (0.86, 1.08)	1.03 (0.91, 1.15)	0.88 (0.82, 0.94)	0.94 (0.88, 1.01)
Pre-menopause	0.76 (0.71, 0.81)	0.79 (0.73, 0.85)	0.88 (0.82, 0.95)	0.68 (0.65, 0.71)	0.75 (0.72, 0.78)
**Age at surgical menopause (years)** ^a^					
≤40	1.72 (1.50, 1.98)	1.45 (1.26, 1.68)	1.40 (1.21, 1.62)	1.51 (1.37, 1.66)	1.39 (1.26, 1.53)
41–45	1.20 (1.04, 1.39)	1.14 (0.99, 1.33)	1.15 (1.00, 1.33)	1.12 (1.03, 1.23)	1.09 (1.00, 1.20)
46–50	1	1	1	1	1
51–55	0.99 (0.85, 1.16)	1.07 (0.91, 1.26)	1.08 (0.91, 1.27)	1.04 (0.94, 1.14)	1.08 (0.98, 1.19)
>55	1.15 (0.98, 1.35)	1.14 (0.97, 1.35)	1.14 (0.97, 1.35)	1.05 (0.95, 1.16)	1.05 (0.95, 1.17)
**Reproductive period (years)**					
<30	1.87 (1.74, 2.00)	1.43 (1.33, 1.54)	1.37 (1.27, 1.48)	1.55 (1.48, 1.62)	1.34 (1.28, 1.41)
30–34	1.32 (1.24, 1.40)	1.15 (1.08, 1.23)	1.14 (1.07, 1.21)	1.15 (1.11, 1.19)	1.08 (1.04, 1.12)
35–39	1	1	1	1	1
≥40	1.05 (1.01, 1.11)	1.13 (1.07, 1.19)	1.09 (1.03, 1.15)	1.00 (0.97, 1.03)	0.98 (0.96, 1.01)
**Parity**					
0	1	1	1	1	1
1	1.11 (1.04, 1.19)	0.54 (0.44, 0.68)	0.57 (0.46, 0.71)	0.95 (0.91, 0.99)	0.82 (0.73, 0.93)
2	0.84 (0.79, 0.88)	0.45 (0.36, 0.55)	0.48 (0.39, 0.59)	0.80 (0.77, 0.82)	0.72 (0.64, 0.81)
3	0.96 (0.90, 1.02)	0.45 (0.36, 0.56)	0.48 (0.38, 0.59)	0.83 (0.80, 0.86)	0.72 (0.64, 0.81)
4	1.39 (1.27, 1.52)	0.54 (0.43, 0.68)	0.56 (0.44, 0.70)	0.90 (0.85, 0.96)	0.72 (0.64, 0.82)
≥5	2.50 (2.22, 2.81)	0.72 (0.56, 0.92)	0.71 (0.55, 0.91)	1.12 (1.02, 1.22)	0.81 (0.70, 0.94)
**Age at first birth (years)**					
13–21	1	1	1	1	1
22–25	0.62 (0.58, 0.65)	0.82 (0.78, 0.87)	0.85 (0.80, 0.90)	0.84 (0.81, 0.87)	0.95 (0.91, 0.98)
26–29	0.43 (0.40, 0.45)	0.69 (0.65, 0.74)	0.73 (0.69, 0.78)	0.75 (0.72, 0.78)	0.90 (0.87, 0.94)
≥30	0.42 (0.40, 0.45)	0.73 (0.68, 0.78)	0.78 (0.73, 0.84)	0.73 (0.70, 0.76)	0.90 (0.87, 0.94)
**Age at last birth (years)**					
13–25	1	1	1	1	1
26–29	0.70 (0.66, 0.74)	0.99 (0.92, 1.06)	0.99 (0.92, 1.06)	0.86 (0.83, 0.90)	1.00 (0.96, 1.05)
30–33	0.61 (0.58, 0.65)	1.03 (0.94, 1.12)	1.05 (0.96, 1.14)	0.81 (0.78, 0.84)	1.00 (0.95, 1.06)
≥34	0.60 (0.56, 0.64)	0.98 (0.89, 1.08)	1.02 (0.92, 1.13)	0.78 (0.75, 0.81)	0.99 (0.93, 1.05)
**Miscarriage**					
No	1	1	1	1	1
Yes	1.17 (1.13, 1.22)	1.13 (1.08, 1.18)	1.12 (1.07, 1.17)	1.11 (1.08, 1.14)	1.11 (1.08, 1.14)
**Infertility**					
No	1	1	1	1	1
Yes	0.68 (0.49, 0.95)	0.78 (0.56, 1.10)	0.87 (0.62, 1.22)	0.80 (0.67, 0.96)	0.93 (0.78, 1.11)
**Age of hysterectomy (years)** ^b^					
≤40	1.82 (1.58, 2.08)	1.53 (1.33, 1.76)	1.46 (1.26, 1.68)	1.60 (1.47, 1.76)	1.48 (1.35, 1.62)
41–45	1.25 (1.07, 1.45)	1.19 (1.02, 1.38)	1.20 (1.03, 1.40)	1.14 (1.04, 1.25)	1.11 (1.01, 1.22)
46–50	1	1	1	1	1
51–55	0.98 (0.83, 1.15)	1.05 (0.88, 1.24)	1.07 (0.90, 1.26)	1.03 (0.94, 1.14)	1.07 (0.97, 1.19)
>55	1.10 (0.93, 1.30)	1.16 (0.97, 1.38)	1.18 (0.99, 1.40)	1.02 (0.92, 1.13)	1.04 (0.93, 1.16)
**MHT**					
No	1	1	1	1	1
Yes	1.40 (1.35, 1.46)	1.42 (1.36, 1.48)	1.23 (1.17, 1.30)	1.49 (1.45, 1.52)	1.33 (1.29, 1.36)

MHT, menopausal hormone therapy.

No-frailty was taken as the reference (0) and frailty as event (1) in models.

a 19 234 women who experienced surgical menopause were involved.

b 18 921 women who experienced hysterectomy were involved.

Model 1: age at baseline was adjusted. Model 2: confounders (race, education level, income level, Townsend index of deprivation, cigarette smoking, alcohol drinking, leisure activities, and MHT) were adjusted based on Model 1. Model 3: cardiovascular disease (CVD), hypertension, and diabetes were adjusted based on Model 2. Only model 1 and model 2 were included in the associations with comprehensive frailty, as CVD, hypertension, and diabetes were components of comprehensive frailty. In addition, reproductive factors were adjusted for each other in the last model. For instance, when analyzing the relationship between age at menopause and frailty, age at menarche, and number of live births were further adjusted.

### Age at natural menopause, MHT use, and frailty

Compared to women with natural menopause at age 46–50 years, women with premature (≤40 years) and early (41–45 years) menopause had increased odds of physical frailty, with ORs (95% CI) of (1.32, 1.17–1.48) and (1.09, 1.00–1.18), respectively. Premature menopause (1.32, 1.22–1.42) and early menopause (1.10, 1.04–1.15) were also linked to higher odds of having comprehensive frailty (Table 3). A dose–response relationship showed a negative relationship between age at natural menopause and physical frailty (Fig. 2B) and comprehensive frailty (Fig. 3B). Similar results were observed when the analyses were stratified by race (Supplementary Fig. S1) and age (Supplementary Table S5).

**Figure 4.**
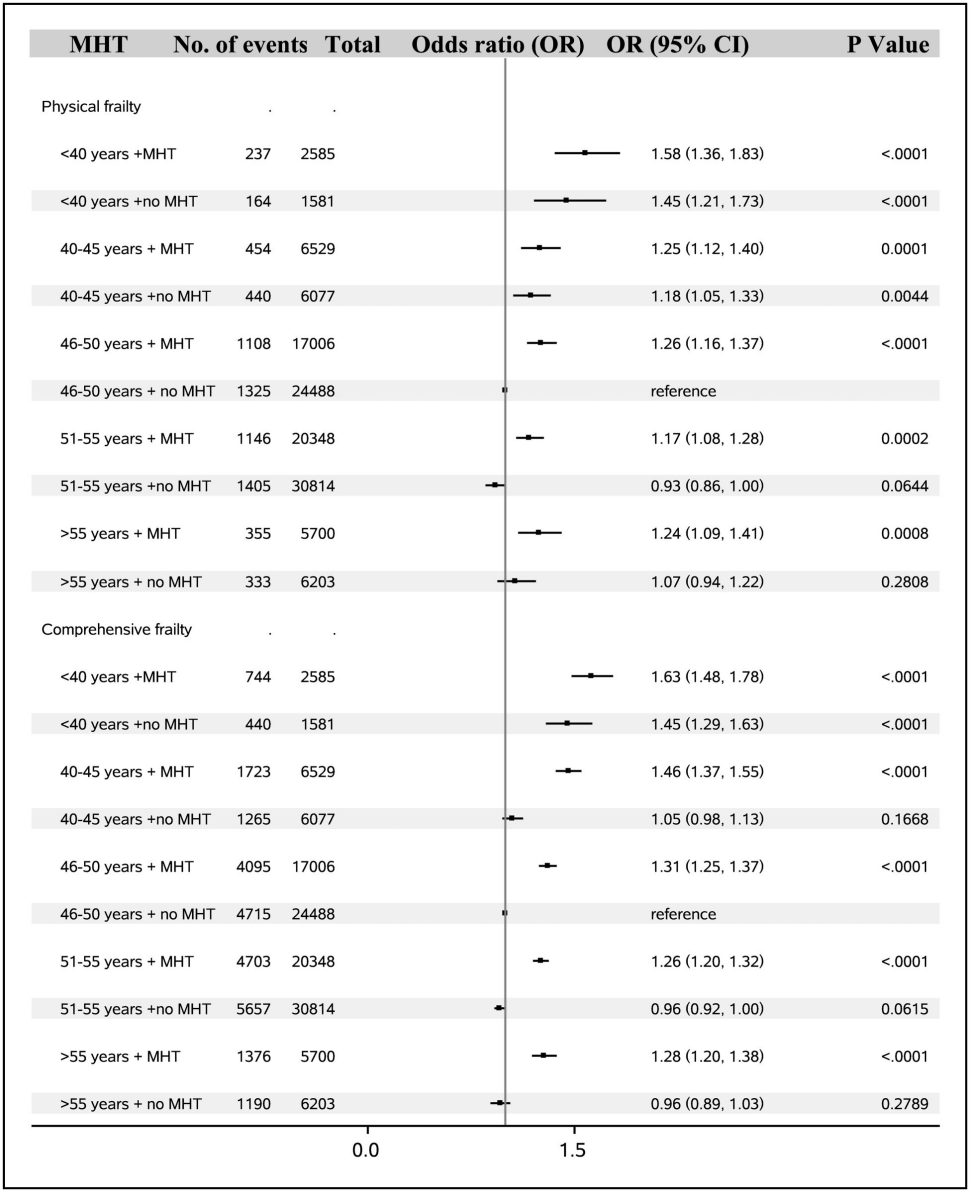
**Combined effect of age at natural menopause and menopause hormone therapy on risk of frailty in women in the UK Biobank (N = 127 139)**. MHT, menopause hormone therapy.

MHT use was linked to higher odds of physical frailty (1.23, 1.17–1.30) and comprehensive frailty (1.33, 1.29–1.36) (Table 3). When the combined effects of age at natural menopause and MHT use on frailty were analyzed, we found that in women with premature (<40 years) or early menopause (40–45 years), MHT use did not increase the odds of physical frailty, while in women with normal menopause (46–50 years and 51–55 years) MHT use was related to increased odds of physical frailty. As to comprehensive frailty, taking MHT was linked to elevated odds of comprehensive frailty in women with menopause after age 40 years (Fig. 4).

### Surgical menopause, MHT use, and frailty

Compared with natural menopause, surgical menopause was associated with 34% higher odds of physical frailty (1.34, 1.27–1.43) and 30% higher odds of comprehensive frailty (1.30, 1.25–1.35). Compared to women with surgical menopause at age 46–50 years, women with surgical menopause before age 40 years and at age 41–45 years were more likely to have frailty, with ORs (95% CI) of 1.40 (1.21–1.62) and 1.15 (1.00–1.33), respectively, for physical frailty, and 1.39 (1.26–1.53), 1.09 (1.00–1.20), respectively, for comprehensive frailty (Table 3). The dose–response relationship showed a negative relationship between age at surgical menopause and both physical frailty (Fig. 2C) and comprehensive frailty (Fig. 3C). After the analyses were stratified by age, earlier surgical menopause had stronger associations with frailty in women aged 60 years or older (Supplementary Table S5).

**Figure 5.**
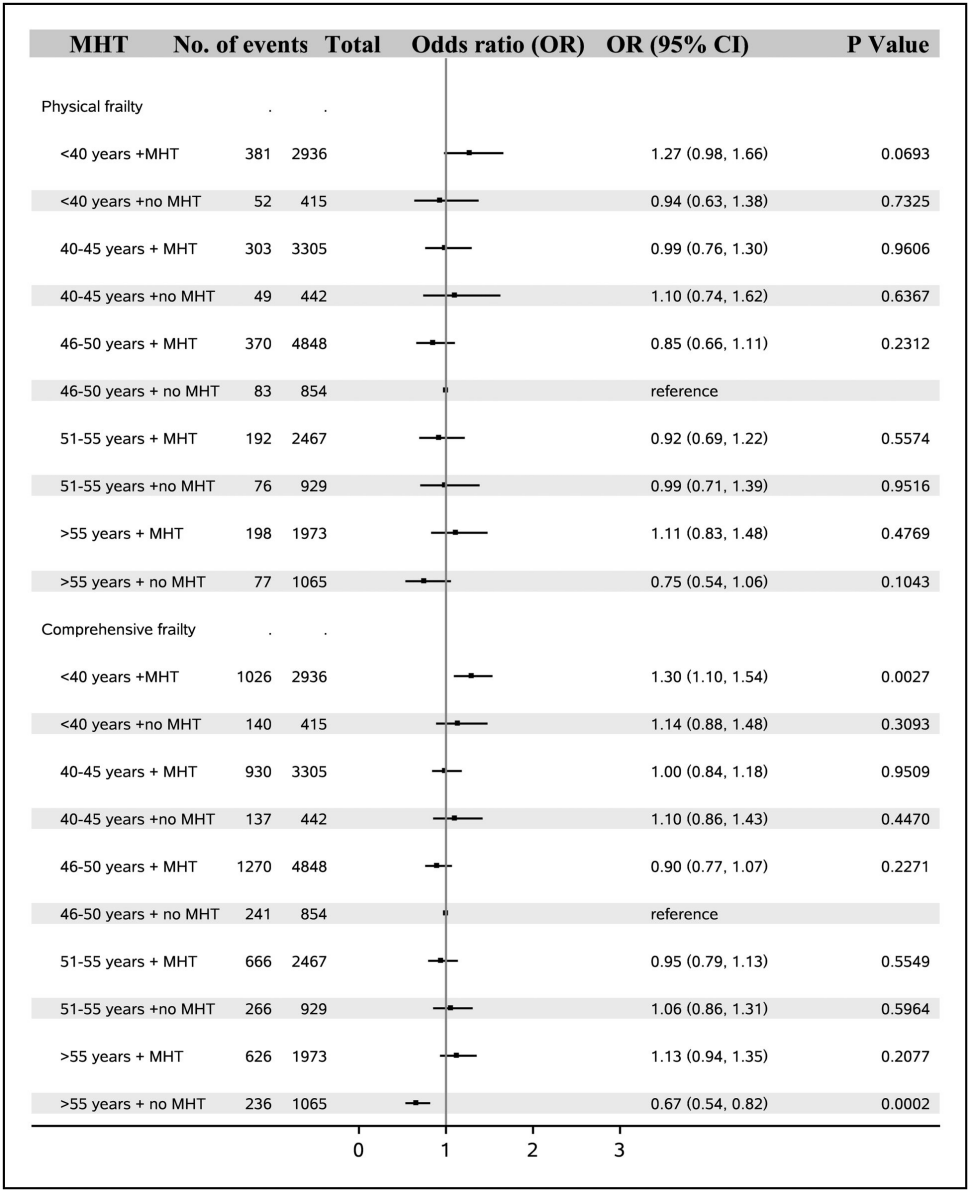
**Combined effect of age at surgical menopause and menopause hormone therapy on risk of frailty in women in the UK Biobank (N = 19 234)**. MHT, menopause hormone therapy.

When the combined effects of age at surgical menopause and MHT use on frailty were analyzed, we did not find that MHT use status modified the association between age at surgical menopause and frailty (Fig. 5).

### Reproductive period and frailty

Compared to women with a reproductive period of 35–39 years, women with shorter (<30 years, 30–34 years) and longer (≥40 years) reproductive periods were all associated with higher odds of physical frailty, with ORs (95% CI) of 1.37 (1.27–1.48), 1.14 (1.07–1.21), and 1.09 (1.03–1.15), respectively. A reproductive period <30 years (1.34, 1.28–1.41) and of 30–34 years (1.08, 1.04–1.12) were also linked to higher odds of having comprehensive frailty (Table 3). Dose–response relationship curves were consistent with the trend when categorical reproductive periods were used (Figs 2D and 3D).

### Number of children, age at births, miscarriage, and frailty

Compared to women who had no children, those who had children showed lower odds of frailty, for example those who had three children were less likely to have physical frailty (0.48, 0.38–0.59) and comprehensive frailty (0.72, 0.64–0.81) (Table 3). Compared to women of a younger age at first birth (13–21 years), those of higher age at first birth had lower odds of physical frailty and comprehensive frailty (Table 3) (Figs 2E and 3E). In addition, experiencing a miscarriage increased the likelihood of physical frailty (1.12, 1.07–1.17) and comprehensive frailty (1.11, 1.08–1.14).

After missing covariates were imputed, findings were consistent with the main analysis (Supplementary Table S6).

## Discussion

### Summary of findings

In this large-scale population-based study, our findings showed that early menarche, at <13 years, age at menopause <45 years, surgical menopause, and a shorter reproductive period <35 years were associated with increased odds of physical frailty and comprehensive frailty, while having two or three children, and a late age at first child decreased the odds of frailty. MHT use was linked to higher physical frailty in women with normal menopause age but not in those with early menopause.

### Age at menarche, age at natural menopause, and frailty

Only one cross-sectional study had investigated the association between age at menarche and frailty (Mamalaki *et al.*, 2019). Including 1148 Greek women of age 65 years or older, Mamalaki *et al.* (2019) found no association between age at menarche and frailty (β = 0.030, *P* = 0.309). Contrary to that study, we observed a J-shape relationship between age at menarche and odds of frailty, i.e. women with both earlier and later age at menarche were related to higher odds of frailty. The average age in the Mamalaki *et al.* (2019) study was 72.2 years, whereas the mean age of women in our study was 56.2 years; this difference may have contributed to the inconsistent findings.

Evidence on the association between age at natural menopause and frailty has been inconsistent (Kojima *et al.*, 2022a,b). A recent systematic review and meta-analysis showed that later menopausal age was associated with a lower risk of physical frailty and comprehensive frailty (pooled OR 0.98, 95% CI 0.96–0.99, *P* < 0.001) (Kojima *et al.*, 2022b). In a prospective study involving 1249 women aged over 60 years in England, Kojima *et al.* (2022a) reported that earlier menopause was associated with a higher risk of physical frailty (OR 1.90, 95% CI 1.28–2.81, *P* = 0.001), but no association was found with late menopause. Consistent with previous studies, we also found that early menopause was associated with high odds of frailty. Studies have shown that there was a linear relationship between age at menopause and risk of CVD (Zhu *et al.*, 2019), and late age at menopause was positively associated with obesity-mediated hypertension (Morimoto and Ichihara, 2023). These might explain the J-shape dose–response relationship of menopausal age with physical frailty in our study.

### Surgical menopause and frailty

A recent systemic review reported no association between surgical menopause and physical frailty and comprehensive frailty (Kojima *et al.*, 2022b), although the review only included two observational studies (Huang *et al.*, 2018; Kojima *et al.*, 2022a). One study from England, including 278 women, found that surgical menopause might be linked to elevated risk of physical frailty, although the 95% CI crossed one (OR 1.27, 95% CI 0.82–1.97) (Kojima *et al.*, 2022a). In American women, Huang *et al.* (2018) found that surgical menopause did not carry a greater risk of frailty compared with natural menopause; however, this study may have misclassified participants as robust or prefrail because they were missing a later follow-up and this potentially biased their results toward the null. We observed that women with surgical menopause had around 30% greater likelihood of frailty than those with natural menopause. Owing to the lack of information on causes of surgical menopause, we could not assess the relationship between surgical menopause caused by different reasons and frailty. Given that surgical menopause poses a greater odds of frailty compared to natural menopause, prophylactic bilateral oophorectomy at the time of hysterectomy should be adopted with caution, especially in women with benign conditions and younger than 50 years.

Multiple mechanisms (such as menopause, reproductive duration) might underly the relationship between endogenous estrogen exposure and frailty. Postmenopausal estrogen decline contributes to increased bone loss (Khosla and Pacifici, 2021), muscle weakness (known as sarcopenia) (Messier *et al.*, 2011; Buckinx and Aubertin-Leheudre, 2022), a higher risk of CVD (Zhu *et al.*, 2019), metabolic dysregulation (Mauvais-Jarvis *et al.*, 2013; Jeong and Park, 2022), heightened inflammation (Abildgaard *et al.*, 2020; Khalafi *et al.*, 2021), and cognitive functions (Hao *et al.*, 2023), all of which contribute to frailty.

### MHT use and frailty

The association between MHT status and frailty has been inconsistent. One study found that, compared to women who did not use MHT or used MHT for a short period of time, long-term (≥13 months) use of MHT was related to reduced risk of sarcopenia (OR 0.60, 95% CI 0.41–0.88, *P* = 0.01) (Kim and Kim, 2020). Also, using the same data, the study showed that 2–5 years of MHT use, and initiation of MHT between 50 and 59 years were both related to a lower risk of frailty (*P* < 0.05) (Kim and Lee, 2022). In contrast, evidence from clinical trials did not find MHT use had a protective effect on frailty (Brown *et al.*, 1997; Bemben and Langdon, 2002; Greenspan *et al.*, 2005; Michael *et al.*, 2010) or even led to opposite findings (Verschoor and Tamim, 2019). A randomized controlled trial reported that MHT failed to provide overall protection against functional decline for postmenopausal women aged 65 years or older (Michael *et al.*, 2010). Also, in two other randomized controlled trials, compared to women who did not use MHT, MHT use did not increase muscle strength and muscle size (Brown *et al.*, 1997; Bemben and Langdon, 2002). As a complement to previous research, after combining age at natural menopause and MHT status, we found MHT use could increase the odds of physical frailty in women with normal age at natural menopause after age 45 years, while elevated odds were not observed in women with early natural menopause taking MHT.

### Reproductive period, number of children, and frailty

Consistent with previous studies, we found that a shorter reproductive period (e.g. <30 years) was associated with increased odds of frailty. In Greek women, Mamalaki *et al.* (2019) found that a longer reproductive period was associated with lower risk of comprehensive frailty (β=−0.070, *P* = 0.021). Similarly, in Korean women, the prevalence of physical frailty decreased 4% per annum with increasing reproductive years (*P* = 0.019) (Lee *et al.*, 2020).

The association between number of children and frailty has been unclear (Gomes *et al.*, 2018; Kojima *et al.*, 2020; Taci *et al.*, 2023). Two observational studies also reported that having more children was associated with a higher risk of frailty (Kojima *et al.*, 2020; Taci *et al.*, 2023). However, Andre Hajek found that having more children was associated with a lower risk of slowness and poor grip strength (Hajek and König, 2022), two of the indices for physical frailty. In our study, we observed that having two or three children was associated with 50% lower odds of physical frailty and 25% lower odds of comprehensive frailty. Compared to previous studies, our study accounted for a broader range of potential confounders, particularly the interplay between individual reproductive factors. The connection between more children and reduced frailty in older women could be related to the enhanced social support and social interaction in their later life, which may delay the progression of frailty (Ding *et al.*, 2017; Hajek and König, 2022).

### Age at births and frailty

Gomes *et al.* (2018) had examined the relationship between age at first birth and frailty and found that early birth age (before age 20 years) was associated with an increased odds of frailty (OR 2.15, 95% CI 1.24–3.72). A recent cross-sectional study that included 10 828 women (age ≥45 years) from National Health and Nutrition Examination Survey (NHANES) also reported early birth age (≤26 years) was associated with a high likelihood of frailty in middle-aged and older women (Guo *et al.*, 2023). There has been no research on the relationship between age at last birth and frailty. Our study found that later age at first birth was related to lower odds of frailty. Childbearing at a young age has been linked to low educational attainment, low occupational opportunities, and low incomes, which may expose young women to cumulative disadvantages and, subsequently, to harmful health behaviors across the life course.

### Strengths and limitations

The strengths of this study include the large sample size, which enabled us to examine the associations between comprehensive reproductive factors through the life course and the two types of frailty (physical frailty and comprehensive frailty). Furthermore, the frailty metrics of FP and FI used in this study had been validated (Hanlon *et al.*, 2018; Williams *et al.*, 2019), which enhances the reliability of our findings. There are several limitations in our study. First, the reproductive factors were self-reported and the data might be subject to recall bias. Nevertheless, it has been shown that self-reported reproductive history is accurate (kappa* *=* *0.48–0.50) (Ellison *et al.*, 2000). Second, we lacked information on types and initiation time of MHT. Thus, the detailed combined effect of menopausal age and MHT use on frailty could not be evaluated. Third, we could not identify infertile women who later became pregnant. The relationship between these women and those with permanent infertility and frailty may be quite different. The number of infertile women in our study may be underestimated. Also, individuals who participated in the UK Biobank are relatively healthier (e.g. lower rates of smoking), well-educated, and less deprived than the general UK population. This limited the generalization of our findings to the whole population. Finally, owing to the nature of a cross-sectional study, some covariates occurred in time after the reproductive history variables, such as the disease variables CVD, hypertension, and diabetes. These variables might be mediators rather than confounders, and the association between reproductive history variables and frailty might be underestimated.

In conclusion, reproductive factors experienced by women throughout their life course play a significant role in a woman’s overall health and well-being, including the likelihood of frailty in later life. Identifying these reproductive factors as potential predictors of frailty can inform healthcare providers and policymakers about the importance of considering a woman’s reproductive history when assessing their risk for frailty. Further research is needed to fully understand the underlying mechanisms and to develop targeted interventions that address the specific needs of women at risk for frailty.

## Supplementary Material

hoae038_Supplementary_Data

## Data Availability

The data described in the manuscript will be made available for researchers who apply to use the UK Biobank data set by registering and applying at https://www.ukbiobank.ac.uk/enable-your-research/register.
