# Supplementary material for: Reproductive factors and their association with physical and comprehensive frailty in middle-aged and older women: a large-scale population-based study
Source: Hum Reprod Open. 2024 Jun 14;2024(3):hoae038. doi: 10.1093/hropen/hoae038 (PMC11211215; doi:10.1093/hropen/hoae038)
Supplement: hoae038_Supplementary_Data [file hoae038_supplementary_data.zip › Supplementary Tables final.docx]

| **Supplementary Table S1.** The five frailty criteria for construction of frailty phenotype. | | | |
| --- | --- | --- | --- |
| **Item** | **UKB fields** | **Question/Description** | **Score** |
| **Weight loss** | 2306 | Weight change compared with 1 year ago | Yes, lost weight=1; other=0 |
| **Exhaustion** | 2080 | Frequency of tiredness/lethargy in last weeks | More than half the days or nearly every day=1; other=0 |
| **Slow walking speed** | 924 | Usual walking pace | Slow=1; other=0 |
| **Low physical activity** | 6164 | Types of physical activity in last 4 weeks | None or light activity with a frequency of once per week or less=1;  medium or heavy activity, or light activity more than once per week=0 |
|  | 2624 | Frequency of heavy DIY in last 4 weeks |  |
|  | 1011 | Frequency of light DIY in last 4 weeks |  |
|  | 3637 | Frequency of other exercises in last 4 weeks |  |
|  | 991 | Frequency of strenuous sports in last 4 weeks |  |
|  | 971 | Frequency of walking for pleasure in last weeks |  |
| **Low grip strength** | 46 | Hand grip strength(left) | sex and BMI adjusted cutoffs: Men:  ≤29 kg for BMI ≤24;  ≤30 kg for BMI 24.1-26;  ≤30 kg for BMI 26.1-28;  ≤32 kg for BMI >28 Women:  ≤17 kg for BMI ≤23;  ≤17.3 kg for BMI 23.1-26;  ≤18 kg for BMI 26.1-29;  ≤21 kg for BMI>29 |
|  | 47 | hand grip strength (right) |  |
|  | 31 | sex |  |
|  | 21001 | BMI (kg/m^2^) |  |
| The scoring method for physical frailty phenotype (FP) is adapted from Fried^1^ and Hanlon^2^, et al. UKB: UK Biobank  ^1^Fried LP, Tangen CM, Walston J, et al. Frailty in older adults: evidence for a phenotype. J Gerontol A Biol Sci Med Sci 2001; 56(3): M146-56.  ^2^Hanlon P, Nicholl BI, Jani BD, Lee D, McQueenie R, Mair FS. Frailty and pre-frailty in middle-aged and older adults and its association with multimorbidity and mortality: a prospective analysis of 493 737 UK Biobank participants. Lancet Public Health 2018; 3(7): e323-e32. | | | |

| **Supplementary Table S2.** The frailty items and scoring for construction of the frailty index. | | | | |
| --- | --- | --- | --- | --- |
| **Type of deficit** | **ID** | **Item** | **UKB Fields** | **Score** |
| **Cancer** | 1 | Any cancer diagnosis* | 134, 2453 | No=0; Yes=1 |
|  | 2 | Multiple cancers diagnosed | 134 | No/single=0;  Multiple cancer=1 |
| **Sensory** | 3 | Glaucoma* | 20002 (1277) | No=0; Yes=1 |
|  | 4 | Cataracts | 20002 (1278) | No=0; Yes=1 |
|  | 5 | Hearing difficulty | 2247 | No=0; Yes/completely deaf=1 |
| **Cranial** | 6 | Migraine* | 20002 (1265), 2473 | No=0; Yes=1 |
| **Mental wellbeing** | 7 | Fatigue: frequency of tiredness/lethargy in last two weeks | 2080 | Not at all=0;  Several days=0.25; More than half=0.5;  Nearly every day=1 |
|  | 8 | Self-rated health | 2178 | Excellent=0;  Good=0.25;  Fair=0.5;  Poor=1 |
|  | 9 | Sleep: experience of sleeplessness/insomnia | 1200 | Never/rarely=0; Sometimes=0.5; Usually=1 |
|  | 10 | Depressed feelings: frequency in last two weeks | 2050 | Not at all=0;  Several days=0.25; More than half=0.5;  Nearly every day=1 |
|  | 11 | Self-described nervous personality | 1970 | No=0; Yes=1 |
|  | 12 | Severe anxiety/ panic attacks* | 20002 (1287), 2473 | No=0; Yes=1 |
|  | 13 | Common to feel loneliness | 2020 |  |
|  | 14 | Sense of misery | 1930 | No=0; Yes=1 |
| **Infirmity** | 15 | Infirmity: long-standing illness or disability | 2188 | No=0; Yes=1 |
|  | 16 | Falls in last year | 2296 | No falls=0;  One fall=0.5;  More than one=1 |
|  | 17 | Fractures/broken bones in last five years | 2463 | No=0; Yes=1 |
|  | 18 | Pneumonia* | 20002 (1398), 2473 | No=0; Yes=1 |
|  | 19 | Chronic bronchitis/  emphysema* | 20002 (1113), 6152 | No=0; Yes=1 |
|  | 20 | Asthma* | 20002 (1111), 6152 | No=0; Yes=1 |
| **Cardiometabolic** | 21 | Diabetes* | 20002 (1220,1223), 2443 | No=0; Yes=1 |
|  | 22 | Myocardial infarction* | 20002 (1075), 6150 | No=0; Yes=1 |
|  | 23 | Angina* | 20002 (1074), 6150 | No=0; Yes=1 |
|  | 24 | Stroke* | 20002 (1081,1583), 6150 | No=0; Yes=1 |
|  | 25 | High blood pressure* | 20002 (1065), 6150 | No=0; Yes=1 |
|  | 26 | Hypothyroidism* | 20002 (1226), 2473 | No=0; Yes=1 |
|  | 27 | Deep-vein thrombosis* | 20002 (1094), 6152 | No=0; Yes=1 |
|  | 28 | High cholesterol* | 20002 (1473), 6153, 6177 | No=0; Yes=1 |
| **Musculoskeletal** | 29 | Rheumatoid arthritis* | 20002 (1464), 2473 | No=0; Yes=1 |
|  | 30 | Osteoarthritis* | 20002 (1465), 2473 | No=0; Yes=1 |
|  | 31 | Gout* | 20002 (1466), 2473 | No=0; Yes=1 |
|  | 32 | Osteoporosis* | 20002 (1309), 2473 | No=0; Yes=1 |
| **Immunological** | 33 | Hay fever/allergic rhinitis  or eczema* | 20002 (1387/1452), 6152 | No=0; Yes=1 |
|  | 34 | Psoriasis* | 20002 (1453), 2473 | No=0; Yes=1 |
| **Gastrointestinal** | 35 | Gastric reflux* | 20002 (1138), 2473 | No=0; Yes=1 |
|  | 36 | Hiatus hernia* | 20002 (1474), 2473 | No=0; Yes=1 |
|  | 37 | Gall stones* | 20002 (1162), 2473 | No=0; Yes=1 |
|  | 38 | Diverticulitis* | 20002 (1458), 2473 | No=0; Yes=1 |

Variables included in frailty index were adapted from Williams^3^ et al. Other criteria for definition of fraility items are shown elswhere by Mutz^4^, et al.

* Participants reported medically diagnosed conditions for these items. For items with multiple corresponding data fields, we first coded “Yes” and “No” responses using the self-report items and, including for data field 2473. Additional “Yes” responses were then ascertained from the nurse led interview diagnostic codes (data field 20002). Individuals with missing data for both data fields 2473 and 20002 were coded as missing data.

^3^Williams DM, Jylhävä J, Pedersen NL, Hägg S. A Frailty Index for UK Biobank Participants. J Gerontol A Biol Sci Med Sci 2019; 74(4): 582-7.

^4^Mutz J, Choudhury U, Zhao J, Dregan A. Frailty in individuals with depression, bipolar disorder and anxiety disorders: longitudinal analyses of all-cause mortality. BMC Med 2022; 20(1): 274.

UKB: UK Biobank

| **Supplementary Table S3.** Exposures and covariates definitions and descriptions. | | | |
| --- | --- | --- | --- |
| **Variables** | **Categorizations** | **UK Biobank Code** | **Descriptions** |
| **Age at menarche** | <11 years,  12 years,  13 years,  14 years,  15 years,  ≥16 years | 2714 | Touchscreen question  "How old were you when your periods started? " |
| **Number of children** | 0,  1,  2,  3,  4,  ≥5 | 2734 | Touchscreen question  "How many children have you given birth to? (Please include live births only)" |
| **Age at first birth** | 13-21 years,  22-25 years,  26-29 years,  ≥30 years, | 2754, 3872 | Touchscreen question   1. "How old were you when you had your FIRST child?"   Touchscreen question   1. "How old were you when you had your child?" |
| **Age at last birth** | 13-25 years,  26-29 years,  30-33 years,  ≥34 years, | 2764, 3872 | Touchscreen question   1. "How old were you when you had your LAST child?"   Touchscreen question   1. "How old were you when you had your child?" |
| **Age at natural menopause** | ≤40 years,  41-45 years,  46-50 years,  51-55 years,  >55 years, | 3581, 2724 | Touchscreen question   1. "How old were you when your periods stopped?" 2. "Have you had your menopause (periods stopped)?" |
| **Age at surgical menopause** | ≤40 years,  41-45 years,  46-50 years,  51-55 years,  >55 years, | 3882 | Touchscreen question "How old were you when you had BOTH ovaries removed?" |
| **Age of hysterectomy** | ≤40 years,  41-45 years,  46-50 years,  51-55 years,  >55 years, | 2824 | Touchscreen question "How old were you when you had your hysterectomy?" |
| **Perimenopause** | Yes or NO | 3700,3710, 3720 | Touchscreen question  (i)"How many days since your last menstrual period?"  (ii) "How many days is your usual menstrual cycle? (The number of days between each menstrual period)"  (iii) "Are you menstruating today? (We are asking this as it may affect the urine sample that you have been asked to provide)" |
| **Premenopause** | Yes or NO | 3700,3710,3720,2724 | Touchscreen question  (i)"How many days since your last menstrual period?"  (ii) "How many days is your usual menstrual cycle? (The number of days between each menstrual period)"  (iii) "Are you menstruating today? (We are asking this as it may affect the urine sample that you have been asked to provide)"  (iv) "Have you had your menopause (periods stopped)?" |
| **Miscarriage** | Yes or NO | 2774 | Touchscreen question  "Have you ever had any stillbirths, spontaneous miscarriages or terminations?" |
| **Infertility** | Yes or NO | ICD-10 N97 | Female infertility |
| **Age (years)** | Age at baseline | 21022 | Date attended baseline assessment minus date of birth |
| **Ethnicity** | White,  Non-white | 21000 | Touchscreen questionnaire: “What is your ethnic group?” |
| **Education levels** | Years of education  ≤10,  11-12,  ＞12 | 6138, 845 | Touchscreen questionnaire:  (i) “which of the following qualifications do you have?”  (ii) “At what age did you complete your continuous full-time education?” |
| **Income levels** | Level1: Less than £18000,  Level2: £18000 to 30999,  Level3: £31,000 to £51,999,  Level 4: Greater than £52,000 | 738 | Touchscreen questionnaire: “what is the average total income before tax received by your HOUSEHOLD? |
| **BMI (kg/m^2^)** | Underweight<18.5,  Normal (18.5, 24.9),  Overweight (25.0, 29.9),  Obese≥30.0 | 21001 | Physical examination: body mass index |
| **Smoking status** | Never,  Former,  Current | 20116 | Touchscreen questionnaire: “how often do you smoke tobacco?” |
| **Alcohol intake** | Daily or almost daily,  3-4 times a week,  1-2 times a week,  Occasionally,  Never | 1558 | Touchscreen questionnaire: “About how often do you drink alcohol?” |
| **Leisure/social activities** | Sports club or gym,  Pub or social club,  Religious group,  Adult education class,  Other group activity,  None of the above,  Prefer not to answer | 6160 | Touchscreen question "Which of the following do you attend once a week or more often? (You can select more than one)" |
| **Menopausal hormone therapy (MHT)** | Yes,  No | 2814 | Touchscreen question "Have you ever used hormone replacement therapy (HRT)?" |
| **CVD** | Yes,  No | 6150 | Touchscreen question "Has a doctor ever told you that you have had any of the following conditions? (You can select more than one answer)" |
| **Hypertension** | Yes,  No | 6153 | Touchscreen question "Do you regularly take any of the following medications? (You can select more than one answer)" |
| **Diabetes status** | Yes,  No | 2443,6153 | Touchscreen question  (i) Has a doctor ever told you that you  have diabetes? (include those who only had diabetes during pregnancy)  (ii) Do you regularly take any of the following medications? (You can select more than one answer)" |

CVD: cardiovascular disease

| **Supplementary Table S4.** Baseline characteristics of women who were included and excluded. | | |
| --- | --- | --- |
|  | **Included (n=189 898)** | **Excluded (n=83 414)** |
| **Age at baseline (years)** | 56.2±7.9 | 56.7±8.1 |
| **Race/ethnicity** |  |  |
| White | 180 789 (95.2) | 76 587 (91.8) |
| Asian | 3289 (1.7) | 2285 (2.7) |
| Black | 2595 (1.4) | 2057 (2.5) |
| Other | 3225 (1.7) | 2485 (3.0) |
| **Education level (years)** |  |  |
| <=10 | 91 152 (48.0) | 45 457 (55.6) |
| 11-12 | 24 150 (12.7) | 9957 (12.2) |
| >12 | 74 596 (39.3) | 26 403 (32.2) |
| **Income level (£)** |  |  |
| <18 000 | 45 333 (23.9) | 23 203 (28.2) |
| 18 000-30 999 | 47 576 (25.1) | 20 685 (25.1) |
| 31 000-51 999 | 48 529 (25.5) | 19 675 (23.9) |
| >=52 000 | 48 460 (25.5) | 18 742 (22.8) |
| **Townsend deprivation index** |  |  |
| Q1 | 48 797 (25.7) | 19 557 (23.5) |
| Q2 | 48 235 (25.4) | 20 026 (24.1) |
| Q3 | 47 585 (25.1) | 20 447 (24.6) |
| Q4 | 45 281 (23.8) | 23 055 (27.8) |
| **BMI** |  |  |
| <18.5kg/m^2^ | 1479 (0.8) | 601 (0.7) |
| 18.5-24.9kg/m^2^ | 76 340 (40.2) | 29 319 (35.8) |
| 25.0-29.9kg/m^2^ | 68 830 (36.3) | 31 034 (37.8) |
| >=30 kg/m^2^ | 43 249 (22.7) | 21 058 (25.7) |
| **Alcohol drinking** |  |  |
| Never drinker | 9980 (5.3) | 5998 (7.3) |
| Former drinker | 6467 (3.4) | 3512 (4.3) |
| Current drinker | 173 451 (91.3) | 73 101 (88.4) |
| **Cigarette smoking** |  |  |
| Never smoker | 113 072 (59.6) | 48 960 (59.7) |
| Former smoker | 60 416 (31.8) | 25 067 (30.6) |
| Current smoker | 16 410 (8.6) | 7953 (9.7) |
| **No. of leisure activities** |  |  |
| 0 | 52 490 (27.6) | 25 668 (31.3) |
| 1 | 80 191 (42.2) | 34 779 (42.4) |
| >=2 | 57 217 (30.1) | 21 549 (26.3) |
| **MHT** |  |  |
| No | 119 843 (63.1) | 45 475 (55.5) |
| Yes | 70 055 (36.9) | 36 444 (44.5) |
| **Diabetes status** |  |  |
| No | 183 280 (96.5) | 78 572 (95.5) |
| Yes | 6618 (3.5) | 3684 (4.5) |
| **CVD status** |  |  |
| No | 181 004 (95.3) | 77 902 (93.4) |
| Yes | 8894 (4.7) | 5512 (6.6) |
| **Hypertension status** |  |  |
| No | 143 713 (75.7) | 58 835 (71.5) |
| Yes | 46 185 (24.3) | 23 486 (28.5) |
| Data are n (%). MHT, menopausal hormone therapy, CVD: cardiovascular disease | | |

| **Supplementary Table S5.** Reproductive characteristics and risks of physical frailty and comprehensive frailty stratified by age in women. | | | | | |
| --- | --- | --- | --- | --- | --- |
|  | **Physical frailty** | |  | **Comprehensive frailty** | |
|  | **<60 years** | **≥60 years** |  | **<60 years** | **≥60 years** |
| **Age at menarche (years)** |  |  |  |  |  |
| <11 | 1.43 (1.32, 1.56) | 1.37 (1.25, 1.50) |  | 1.17 (1.11, 1.23) | 1.08 (1.02, 1.14) |
| 12 | 1.13 (1.03, 1.23) | 1.10 (1.00, 1.22) |  | 1.03 (0.98, 1.09) | 1.03 (0.97, 1.08) |
| 13 | 1.04 (0.95, 1.13) | 1.04 (0.94, 1.14) |  | 1.02 (0.97, 1.07) | 1.03 (0.98, 1.09) |
| 14 | 1 | 1 |  | 1 | 1 |
| 15 | 1.03 (0.93, 1.15) | 1.05 (0.93, 1.17) |  | 1.03 (0.97, 1.09) | 1.05 (0.98, 1.12) |
| ≥16 | 1.08 (0.96, 1.23) | 1.19 (1.03, 1.36) |  | 1.16 (1.08, 1.24) | 1.09 (1.01, 1.19) |
| **Menopause type** |  |  |  |  |  |
| Premenopause | 1.23 (0.87, 1.74) | 0.76 (0.73, 0.79) |  | 0.76 (0.73, 0.79) | 0.81 (0.63, 1.05) |
| Perimenopause | 0.87 (0.24, 3.20) | 0.95 (0.89, 1.01) |  | 0.95 (0.89, 1.01) | 1.28 (0.56, 2.94) |
| Natural menopause | 1 | 1 |  | 1 | 1 |
| Surgical menopause | 1.30 (1.20, 1.41) | 1.30 (1.20, 1.41) |  | 1.36 (1.28, 1.44) | 1.26 (1.20, 1.32) |
| **Age at natural menopause (years)** |  |  |  |  |  |
| ≤40 | 1.26 (1.07, 1.49) | 1.39 (1.18, 1.64) |  | 1.41 (1.27, 1.56) | 1.24 (1.12, 1.38) |
| 41-45 | 1.02 (0.91, 1.16) | 1.15 (1.03, 1.29) |  | 1.07 (1.00, 1.15) | 1.12 (1.05, 1.20) |
| 46-50 | 1 | 1 |  | 1 | 1 |
| 51-55 | 0.89 (0.82, 0.97) | 0.95 (0.87, 1.03) |  | 0.97 (0.93, 1.02) | 0.94 (0.94, 0.99) |
| >55 | 1.15 (0.98, 1.35) | 0.99 (0.89, 1.11) |  | 1.01 (0.92, 1.10) | 0.95 (0.89, 1.01) |
| Perimenopause | 0.99 (0.88, 1.12) | 0.83 (0.22, 3.07) |  | 0.95 (0.89, 1.20) | 1.26 (0.55, 2.90) |
| Premenopause | 0.86 (0.79, 0.93) | 1.25 (0.88, 1.77) |  | 0.77 (0.73, 0.80) | 0.81 (0.63, 1.04) |
| **Age at surgical menopause (years)^a^** |  |  |  |  |  |
| ≤40 | 1.24 (1.01, 1.52) | 1.59 (1.28, 1.97) |  | 1.29 (1.13, 1.48) | 1.52 (1.32, 1.75) |
| 41-45 | 1.06 (0.86, 1.30） | 1.23 (0.98, 1.54) |  | 1.10 (0.96, 1.25) | 1.08 (0.94, 1.24) |
| 46-50 | 1 | 1 |  | 1 | 1 |
| 51-55 | 0.90 (0.69, 1.18） | 1.21 (0.98, 1.49) |  | 1.11 (0.95, 1.31) | 1.06 (0.94, 1.20) |
| >55 | 0.92 (0.61, 1.39） | 1.29 (1.06, 1.58) |  | 0.71 (0.54, 0.93) | 1.16 (1.03, 1.30) |
| **Reproductive period (years)** |  |  |  |  |  |
| <30 | 1.35 (1.22, 1.50) | 1.38 (1.24, 1.53) |  | 1.38 (1.29, 1.47) | 1.32 (1.23, 1.40) |
| 30-34 | 1.14 (1.05, 1.25) | 1.13 (1.03, 1.24) |  | 1.10 (1.04, 1.16) | 1.07 (1.02, 1.13) |
| 35-39 | 1.11 (1.03, 1.21) | 1.08 (1.00, 1.16) |  | 0.99 (0.95, 1.04) | 0.97 (0.94, 1.01) |
| ≥40 |  |  |  |  |  |
| **Parity** |  |  |  |  |  |
| 0 | 1 | 1 |  | 1 | 1 |
| 1 | 0.47 (0.35, 0.63) | 0.68 (0.49, 0.94) |  | 0.73 (0.62, 0.86) | 0.88 (0.74, 1.05) |
| 2 | 0.40 (0.30, 0.54) | 0.56 (0.41, 0.77) |  | 0.63 (0.54, 0.74) | 0.79 (0.67, 0.93) |
| 3 | 0.39 (0.29, 0.52) | 0.58 (0.42, 0.80) |  | 0.62 (0.53, 0.73) | 0.80 (0.68, 0.95) |
| 4 | 0.46 (0.33, 0.63) | 0.67 (0.47, 0.94) |  | 0.61 (0.51, 0.73) | 0.82 (0.68, 0.98) |
| ≥5 | 0.60 (0.43, 0.85) | 0.81 (0.56, 1.16) |  | 0.74 (0.60, 0.91) | 0.87 (0.71, 1.07) |
| **Age at first birth (years)** |  |  |  |  |  |
| 13-21 | 1 | 1 |  | 1 | 1 |
| 22-25 | 0.83 (0.76, 0.90) | 0.89 (0.82, 0.97) |  | 0.94 (0.89, 0.99) | 0.96 (0.92, 1.01) |
| 26-29 | 0.67 (0.61, 0.73) | 0.81 (0.74, 0.89) |  | 0.85 (0.81, 0.90) | 0.96 (0.91, 1.01) |
| ≥30 | 0.72 (0.66, 0.79) | 0.88 (0.78, 0.98) |  | 0.85 (0.80, 0.90) | 1.00 (0.94, 1.06) |
| **Age at last birth (years)** |  |  |  |  |  |
| 13-25 | 1 | 1 |  | 1 | 1 |
| 26-29 | 1.03 (0.93, 1.14) | 0.95 (0.86, 1.05) |  | 0.98 (0.92, 1.05) | 1.01 (0.96, 1.07) |
| 30-33 | 1.03 (0.91, 1.17) | 1.05 (0.92, 1.18) |  | 0.95 (0.88, 1.02) | 1.04 (0.97, 1.12) |
| ≥34 | 0.96 (0.84, 1.11) | 1.07 (0.93, 1.23) |  | 0.92 (0.84, 1.00) | 1.04 (0.96, 1.14) |
| **Miscarriage** |  |  |  |  |  |
| No | 1 | 1 |  | 1 | 1 |
| Yes | 1.11 (1.05, 1.17) | 1.13 (1.06, 1.21) |  | 1.09 (1.05, 1.13) | 1.14 (1.09, 1.18) |
| **Infertility** |  |  |  |  |  |
| No | 1 | 1 |  | 1 | 1 |
| Yes | 0.90 (0.64, 1.27) | N/A |  | 0.96 (0.81, 1.15) | 1.19 (0.12, 11.53) |
| **Age of hysterectomy(years)^b^** |  |  |  |  |  |
| ≤40 | 1.35 (1.10, 1.66) | 1.56 (1.26, 1.91) |  | 1.41 (1.23, 1.61) | 1.58 (1.38, 1.80) |
| 41-45 | 1.15 (0.93, 1.42) | 1.25 (1.00, 1.56) |  | 1.12 (0.98, 1.29) | 1.09 (0.95, 1.25) |
| 46-50 | 1 | 1 |  | 1 | 1 |
| 51-55 | 0.93 (0.70, 1.24) | 1.16 (0.93, 1.43) |  | 1.14 (0.96, 1.34) | 1.04 (0.92, 1.18) |
| >55 | 0.89 (0.59, 1.34) | 1.32 (1.07, 1.62) |  | 0.81 (0.63, 1.03) | 1.12 (0.99, 1.27) |

N/A, Not applicable

No-frailty was taken as the reference (0) and frailty as event (1) in models;

^a^19 234 women who experienced surgical menopause were involved;

^b^18 921 women who experienced hysterectomy were involved;

Model 1: age at baseline was adjusted. Model 2: confounders (race, education level, income level, Townsend index of deprivation, cigarette smoking, alcohol drinking, leisure activities, and menopausal hormone therapy) were adjusted based on Model 1. Model 3: cardiovascular disease (CVD), hypertension and diabetes were adjusted based on Model 2. Only model 1 and model 2 were included in the associations with comprehensive frailty, as CVD, hypertension and diabetes were components of comprehensive frailty. In addition, reproductive factors were adjusted for each other in the last model. For instance, when analyzing the relationship between age at menopause and frailty, age at menarche and number of live births were further adjusted.

**Supplementary Table S6.** Odd ratios and 95%CI between female reproductive characteristics and physical frailty and comprehensive frailty (N=194,579).

|  | **Physical frailty** | | |  | **Comprehensive frailty** | |
| --- | --- | --- | --- | --- | --- | --- |
|  | **Model 1** | **Model 2** | **Model 3** |  | **Model 1** | **Model 2** |
| **Age at menarche (years)** |  |  |  |  |  |  |
| <11 | 1.55 (1.46, 1.64) | 1.51 (1.42, 1.60) | 1.41 (1.32, 1.49) |  | 1.25 (1.21, 1.29) | 1.13 (1.09, 1.17) |
| 12 | 1.10 (1.03, 1.17) | 1.14 (1.07, 1.22) | 1.11 (1.04, 1.19) |  | 1.06 (1.02, 1.10) | 1.03 (0.99, 1.07) |
| 13 | 0.99 (0.93, 1.06) | 1.04 (0.98, 1.11) | 1.04 (0.97, 1.10) |  | 1.03 (0.99, 1.07) | 1.03 (0.99, 1.06) |
| 14 | 1 | 1 | 1 |  | 1 | 1 |
| 15 | 1.13 (1.05, 1.22) | 1.04 (0.97, 1.13) | 1.05 (0.97, 1.13) |  | 1.04 (1.00, 1.09) | 1.03 (0.99, 1.08) |
| ≥16 | 1.35 (1.23, 1.47) | 1.15 (1.05, 1.25) | 1.14 (1.04, 1.25) |  | 1.14 (1.09, 1.21) | 1.12 (1.06, 1.18) |
| **Menopause type** |  |  |  |  |  |  |
| Premenopause | 0.77 (0.73, 0.82) | 0.77 (0.73, 0.82) | 0.88 (0.82, 0.93) |  | 0.68 (0.65, 0.70) | 0.75 (0.73, 0.78) |
| Perimenopause | 0.94 (0.85, 1.04) | 0.97 (0.86, 1.08) | 1.02 (0.91, 1.14) |  | 0.87 (0.82, 0.93) | 0.94 (0.88, 1.00) |
| Natural menopause | 1 | 1 | 1 |  | 1 | 1 |
| Surgical menopause | 1.72 (1.63, 1.81) | 1.45 (1.37, 1.53) | 1.34 (1.27, 1.43) |  | 1.52 (1.47, 1.58) | 1.30 (1.26, 1.35) |
| **Age at natural menopause (years)** |  |  |  |  |  |  |
| ≤40 | 1.76 (1.58, 1.96) | 1.37 (1.22, 1.54) | 1.34 (1.19, 1.50) |  | 1.50 (1.39, 1.61) | 1.32 (1.22, 1.41) |
| 41-45 | 1.24 (1.15, 1.34) | 1.11 (1.02, 1.20) | 1.08 (1.00, 1.17) |  | 1.17 (1.11, 1.22) | 1.10 (1.04, 1.15) |
| 46-50 | 1 | 1 | 1 |  | 1 | 1 |
| 51-55 | 0.82 (0.78, 0.87) | 0.92 (0.87, 0.98) | 0.92 (0.87, 0.97) |  | 0.93 (0.90, 0.96) | 0.96 (0.93, 0.99) |
| >55 | 0.95 (0.87, 1.03) | 1.06 (0.97, 1.15) | 1.01 (0.93, 1.11) |  | 0.97 (0.92, 1.02) | 0.97 (0.92, 1.02) |
| Perimenopause | 0.92 (0.82, 1.03) | 0.96 (0.86, 1.08) | 1.01 (0.90, 1.13) |  | 0.88 (0.83, 0.94) | 0.94 (0.88, 1.01) |
| Premenopause | 0.76 (0.71, 0.81) | 0.79 (0.73, 0.85) | 0.87 (0.81, 0.94) |  | 0.68 (0.65, 0.71) | 0.75 (0.72, 0.78) |
| **Age at surgical menopause (years)^a^** |  |  |  |  |  |  |
| ≤40 | 1.70 (1.48, 1.95) | 1.44 (1.25, 1.66) | 1.38 (1.20, 1.60) |  | 1.51 (1.38, 1.65) | 1.39 (1.27, 1.53) |
| 41-45 | 1.20 (1.04, 1.39) | 1.15 (0.99, 1.33) | 1.15 (0.99, 1.33) |  | 1.12 (1.03, 1.22) | 1.09 (0.99, 1.20) |
| 46-50 | 1 | 1 | 1 |  | 1 | 1 |
| 51-55 | 0.97 (0.83, 1.13) | 1.04 (0.88, 1.22) | 1.04 (0.89, 1.23) |  | 1.04 (0.95, 1.15) | 1.08 (0.98, 1.19) |
| >55 | 1.15 (0.99, 1.35) | 1.15 (0.98, 1.36) | 1.15 (0.97, 1.36) |  | 1.05 (0.95, 1.16) | 1.06 (0.95, 1.17) |
| **Reproductive period (years)** |  |  |  |  |  |  |
| <30 | 1.89 (1.77, 2.03) | 1.45 (1.35, 1.56) | 1.38 (1.29, 1.49) |  | 1.55 (1.49, 1.62) | 1.34 (1.28, 1.41) |
| 30-34 | 1.32 (1.25, 1.41) | 1.16 (1.09, 1.23) | 1.15 (1.08, 1.22) |  | 1.15 (1.11, 1.19) | 1.08 (1.05, 1.12) |
| 35-39 | 1 | 1 | 1 |  | 1 | 1 |
| ≥40 | 1.06 (1.00, 1.11) | 1.13 (1.07, 1.19) | 1.09 (1.04, 1.15) |  | 1.00 (0.97, 1.03) | 0.99 (0.96, 1.02) |
| **Parity** |  |  |  |  |  |  |
| 0 | 1 | 1 | 1 |  | 1 | 1 |
| 1 | 1.11 (1.04, 1.19) | 0.53 (0.43, 0.66) | 0.56 (0.45, 0.70) |  | 0.95 (0.91, 0.98) | 0.81 (0.72, 0.91) |
| 2 | 0.83 (0.79, 0.88) | 0.44 (0.36, 0.54) | 0.47 (0.38, 0.58) |  | 0.80 (0.77, 0.82) | 0.71 (0.63, 0.79) |
| 3 | 0.96 (0.90, 1.03) | 0.45 (0.36, 0.55) | 0.47 (0.38, 0.58) |  | 0.83 (0.80, 0.86) | 0.71 (0.63, 0.79) |
| 4 | 1.40 (1.28, 1.53) | 0.53 (0.43, 0.67) | 0.55 (0.44, 0.69) |  | 0.90 (0.85, 0.95) | 0.71 (0.63, 0.80) |
| ≥5 | 2.59 (2.31, 2.91) | 0.72 (0.57, 0.92) | 0.71 (0.55, 0.90) |  | 1.11 (1.01, 1.21) | 0.78 (0.68, 0.91) |
| **Age at first birth (years)** |  |  |  |  |  |  |
| 13-21 | 1 | 1 | 1 |  | 1 | 1 |
| 22-25 | 0.62 (0.59, 0.65) | 0.83 (0.78, 0.87) | 0.86 (0.81, 0.91) |  | 0.84 (0.81, 0.87) | 0.95 (0.91, 0.98) |
| 26-29 | 0.42 (0.40, 0.45) | 0.69 (0.65, 0.73) | 0.73 (0.68, 0.78) |  | 0.75 (0.72, 0.77) | 0.90 (0.87, 0.93) |
| ≥30 | 0.43 (0.40, 0.45) | 0.73 (0.68, 0.78) | 0.79 (0.73, 0.84) |  | 0.73 (0.70, 0.76) | 0.90 (0.86, 0.94) |
| **Age at last birth (years)** |  |  |  |  |  |  |
| 13-25 | 1 | 1 | 1 |  | 1 | 1 |
| 26-29 | 0.70 (0.66, 0.74) | 0.98 (0.92, 1.05) | 0.99 (0.92, 1.06) |  | 0.86 (0.83, 0.89) | 1.00 (0.96, 1.04) |
| 30-33 | 0.62 (0.58, 0.66) | 1.03 (0.94, 1.12) | 1.05 (0.96, 1.14) |  | 0.80 (0.78, 0.84) | 1.00 (0.95, 1.05) |
| ≥34 | 0.60 (0.56, 0.64) | 0.97 (0.88, 1.07) | 1.01 (0.92, 1.12) |  | 0.78 (0.75, 0.81) | 0.98 (0.92, 1.04) |
| **Miscarriage** |  |  |  |  |  |  |
| No | 1 | 1 | 1 |  | 1 | 1 |
| Yes | 1.18 (1.13, 1.22) | 1.13 (1.08, 1.18) | 1.12 (1.07, 1.17) |  | 1.11 (1.08, 1.14) | 1.11 (1.08, 1.14) |
| **Infertility** |  |  |  |  |  |  |
| No | 1 | 1 | 1 |  | 1 | 1 |
| Yes | 0.71 (0.52, 0.99) | 0.82 (0.59, 1.13) | 0.90 (0.65, 1.25) |  | 0.81 (0.69, 0.97) | 0.94 (0.79, 1.12) |
| **Age of hysterectomy (years)^b^** |  |  |  |  |  |  |
| ≤40 | 1.82 (1.59, 2.08) | 1.53 (1.33, 1.76) | 1.46 (1.27, 1.69) |  | 1.61 (1.47, 1.76) | 1.48 (1.35, 1.62) |
| 41-45 | 1.25 (1.08, 1.44) | 1.19 (1.03, 1.38) | 1.21 (1.04, 1.40) |  | 1.14 (1.04, 1.25) | 1.11 (1.01, 1.22) |
| 46-50 | 1 | 1 | 1 |  | 1 | 1 |
| 51-55 | 0.96 (0.81, 1.13) | 1.02 (0.87, 1.21) | 1.04 (0.88, 1.24) |  | 1.04 (0.94, 1.15) | 1.08 (0.98, 1.19) |
| >55 | 1.10 (0.93, 1.30) | 1.16 (0.98, 1.38) | 1.18 (0.99, 1.41) |  | 1.02 (0.92, 1.13) | 1.04 (0.93, 1.15) |

No-frailty was taken as the reference (0) and frailty as event (1) in models;

^a^19 234 women who experienced surgical menopause were involved;

^b^18 921 women who experienced hysterectomy were involved;

Model 1: age at baseline was adjusted. Model 2: confounders (race, education level, income level, Townsend index of deprivation, cigarette smoking, alcohol drinking, leisure activities, and menopausal hormone therapy) were adjusted based on Model 1. Model 3: cardiovascular disease (CVD), hypertension and diabetes were adjusted based on Model 2. Only model 1 and model 2 were included in the associations with comprehensive frailty, as CVD, hypertension and diabetes were components of comprehensive frailty. In addition, reproductive factors were adjusted for each other in the last model. For instance, when analyzing the relationship between age at menopause and frailty, age at menarche and number of live births were further adjusted.
