# Supplementary material for: Reproductive factors and their association with physical and comprehensive frailty in middle-aged and older women: a large-scale population-based study
Source: Hum Reprod Open. 2024 Jun 14;2024(3):hoae038. doi: 10.1093/hropen/hoae038 (PMC11211215; doi:10.1093/hropen/hoae038)
Supplement: hoae038_Supplementary_Data [file hoae038_supplementary_data.zip › Supplementary_Figure_S1_final_EO.docx]

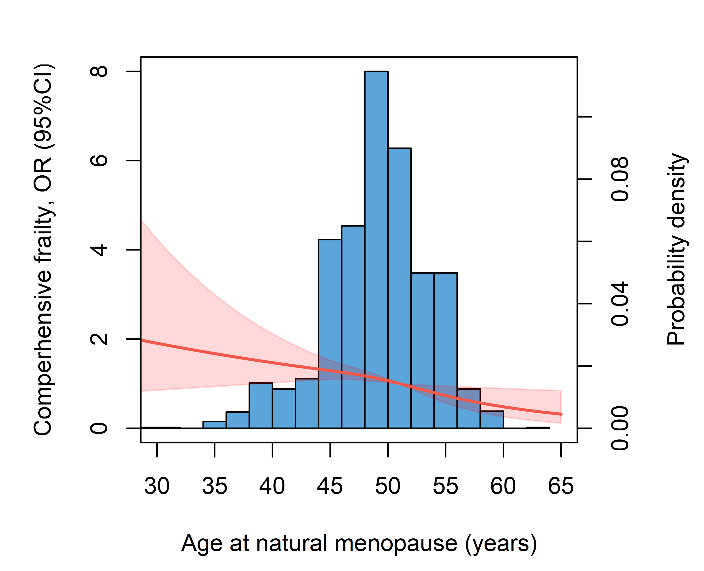

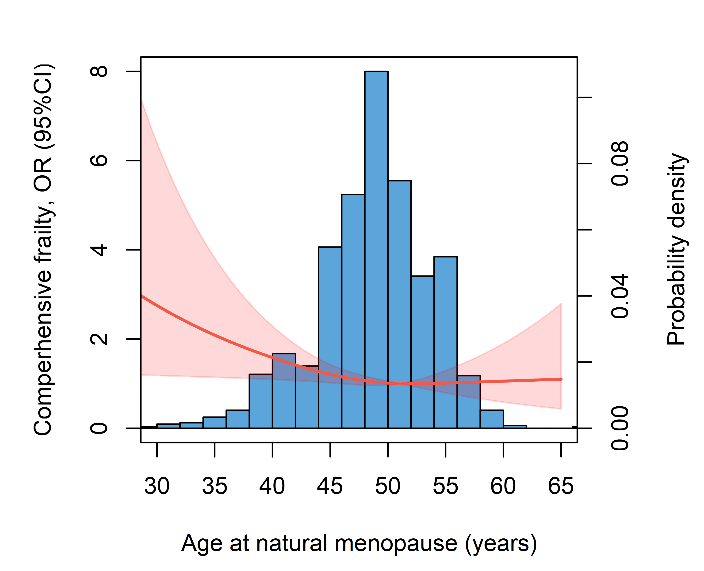

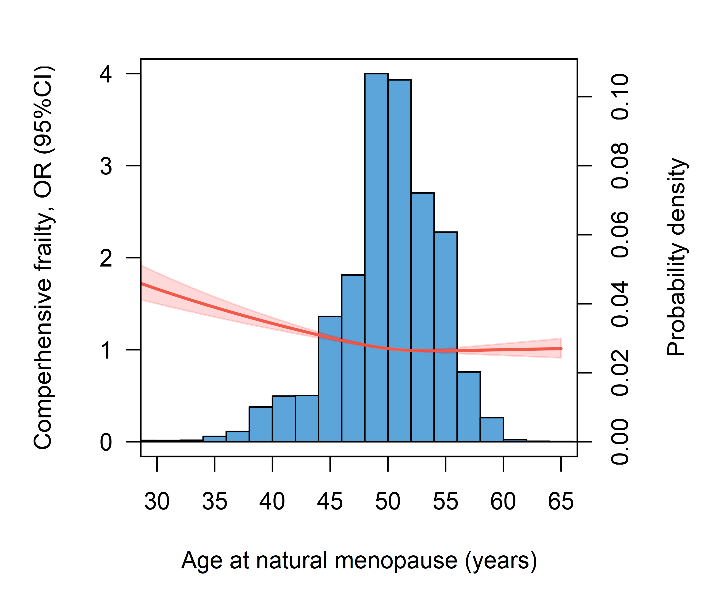

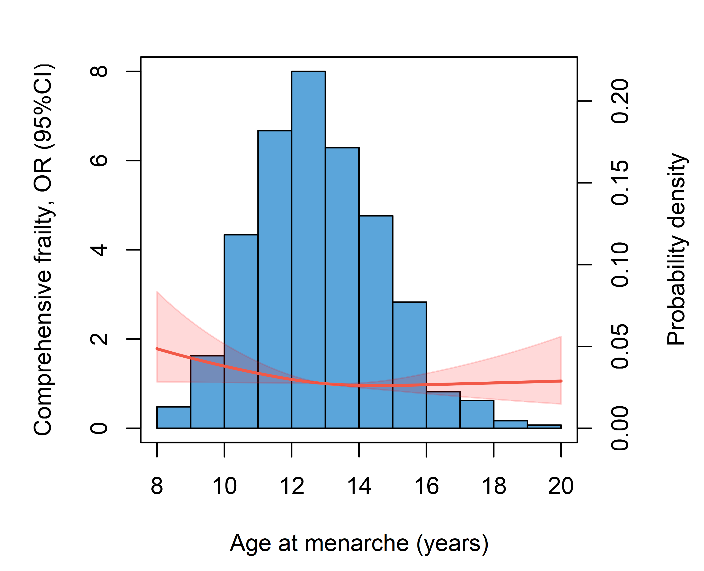

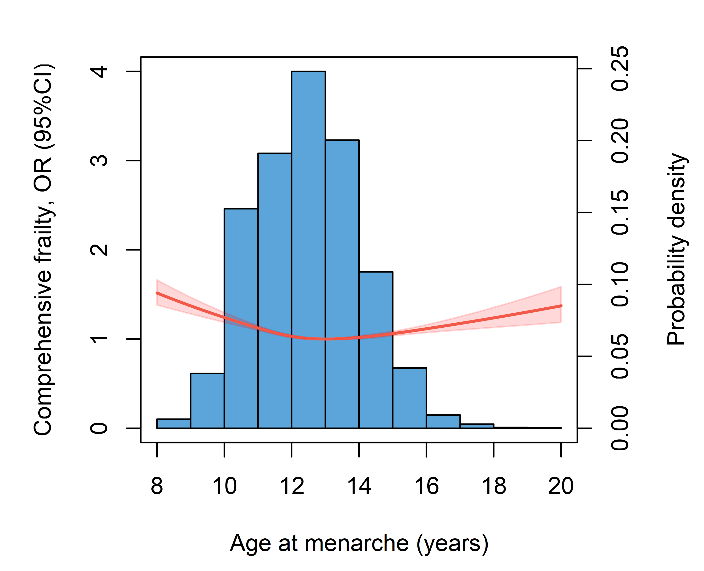

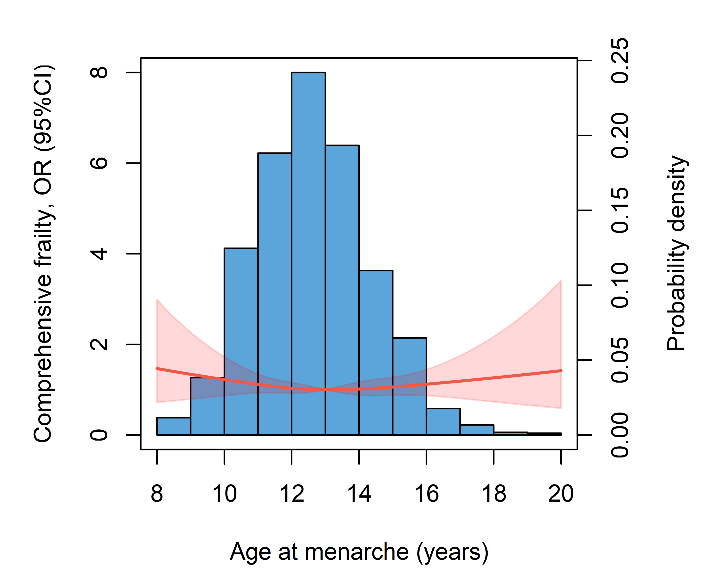


**White**

**White**

**Asian**

**Black**

**Asian**

**Black**


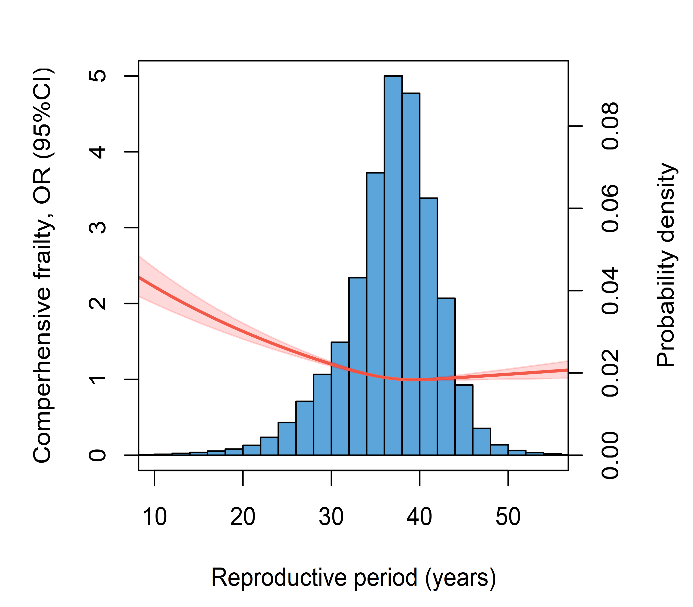

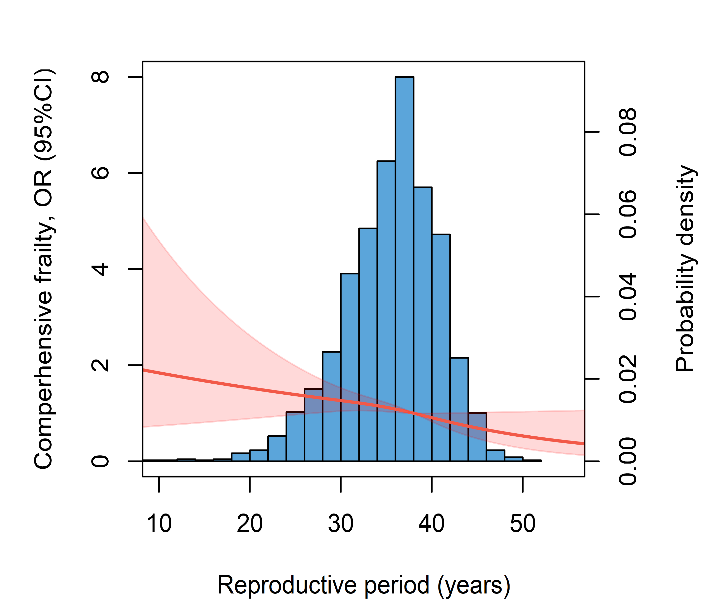

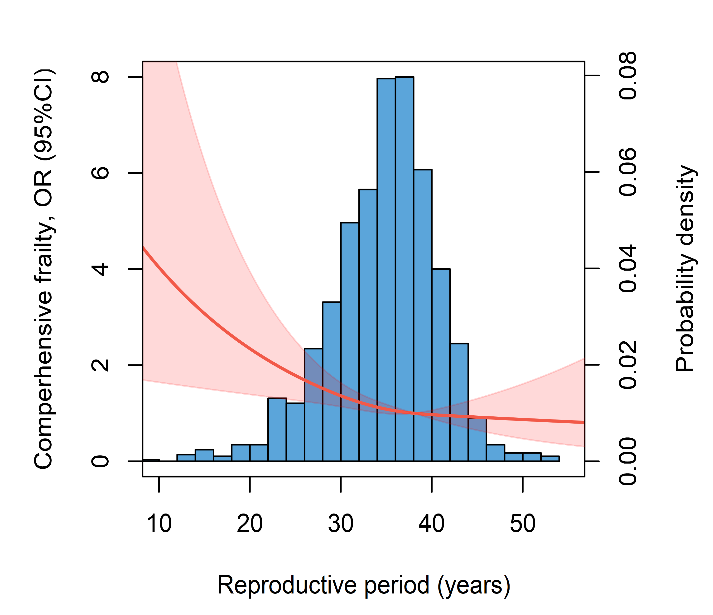


**Asian**

**Black**

**White**

**Supplementary Figure S1.** Reproductive characteristics of women in the UK Biobank and comprehensive frailty, stratified by race.

OR: odds ratio
